# In Parkinson's patient-derived dopamine neurons, the triplication of α-synuclein locus induces distinctive firing pattern by impeding D2 receptor autoinhibition

**DOI:** 10.1186/s40478-021-01203-9

**Published:** 2021-06-07

**Authors:** Min Lin, Phillip M. Mackie, Fatima Shaerzadeh, Joyonna Gamble-George, Douglas R. Miller, Chris J. Martyniuk, Habibeh Khoshbouei

**Affiliations:** 1 Department of Neuroscience, University of Florida, Gainesville, FL 32611 USA; 2 Environmental and Human Toxicology, University of Florida Genetics Institute, Interdisciplinary Program in Biomedical Sciences Neuroscience, College of Veterinary Medicine, University of Florida, Gainesville, FL 32611 USA

**Keywords:** α-synuclein, iPSCs, Dopamine neurons, D2 receptor, Parkinson’s disease

## Abstract

Pathophysiological changes in dopamine neurons precede their demise and contribute to the early phases of Parkinson’s disease (PD). Intracellular pathological inclusions of the protein α-synuclein within dopaminergic neurons are a cardinal feature of PD, but the mechanisms by which α-synuclein contributes to dopaminergic neuron vulnerability remain unknown. The inaccessibility to diseased tissue has been a limitation in studying progression of pathophysiology prior to degeneration of dopamine neurons. To address these issues, we differentiated induced pluripotent stem cells (iPSCs) from a PD patient carrying the α-synuclein triplication mutation (AST) and an unaffected first-degree relative (NAS) into dopaminergic neurons. In human-like dopamine neurons α-synuclein overexpression reduced the functional availability of D2 receptors, resulting in a stark dysregulation in firing activity, dopamine release, and neuronal morphology. We back-translated these findings into primary mouse neurons overexpressing α-synuclein and found a similar phenotype, supporting the causal role for α-synuclein. Importantly, application of D2 receptor agonist, quinpirole, restored the altered firing activity of AST-derived dopaminergic neurons to normal levels. These results provide novel insights into the pre-degenerative pathophysiological neuro-phenotype induced by α-synuclein overexpression and introduce a potential mechanism for the long-established clinical efficacy of D2 receptor agonists in the treatment of PD.

**Supplementary Information:**

The online version contains supplementary material available at 10.1186/s40478-021-01203-9.

## Introduction

Progressive loss of dopaminergic neurons with corresponding increases in α-synuclein characterize many Parkinson's disease (PD) cases [20, 47, 131]. Triplication of the SNCA locus encoding α-synuclein leads to a penetrant form of PD and degeneration of dopamine neurons, suggesting a causal role for α-synuclein in the degeneration process. However, the underlying mechanisms of progressive neuronal loss remain nebulous. Consequently, there is no cure for PD, only therapeutic strategies to alleviate the symptoms of disease. Dopamine replacement strategies are the first choice of treatment and one of the most common pharmacological strategies to sustain the quality of life in PD patients [2, 4, 34, 35, 103, 106, 133]. Specifically, dopamine D2-like receptors (D2R) have historically been one of the primary therapeutic targets. Pre-synaptic D2Rs tightly regulate dopaminergic neuronal activity [11, 19, 23, 37, 56, 90, 96], and previous reports have shown the impairment of D2 receptor-dependent dopaminergic transmission in parkinsonian animal models [74]. In addition, treatment with dopamine D2 receptor agonists restored decreased precursor cell proliferation in parkinsonian animal models [51] and attenuated the compulsive behaviors associated with reduced striatal D2R expression in PD patients [121]. However, despite widespread study in preclinical models and clinical use, the mechanisms underlying D2R-mediated regulation of neuronal activity in the context Parkinson’s Disease—particularly before extensive nigral neuron loss—are not fully understood.

Due to the inaccessibility of diseased tissue, critical investigation into the progression of PD pathophysiology prior to degeneration of dopamine neurons remains improbable. In addition, although several PD animal models have been established, most of these models have failed to reproduce the human disease in its entirety [42, 99, 105]. Recent advances in differentiating human induced pluripotent stem cells (iPSCs) provide a promising tool to investigate the pathological events in human cells that lead to neuronal demise, disease modeling [7, 53], and drug screening [43, 50]. Differentiated iPSCs of parent cells derived from patients harboring disease-related genes have been generated, specifically those related to early onset PD [111, 119], or healthy individuals into the functional dopaminergic neurons [109]. The accessibility of iPSC-derived dopamine neurons from both a PD patient harboring α-synuclein triplication and their unaffected first-degree relative (neurotypical control) provide a disease-relevant system to study how excess α-synuclein leads to neuronal dysfunction. In 2011, the Kunath lab [32] generated sets of iPSC lines from a PD patient with triplication of SNCA, and an unaffected first-degree relative, which serves as a control with a similar genetic background to minimize phenotypic differences not due to the triplication of SNCA [32]. The PD patient-derived cells with α-synuclein triplication are referred to AST cells and the healthy relative-derived cells with normal α-synuclein are referred to NAS cells.

In this study, we differentiated the AST and NAS iPS cell lines to human-like dopamine neurons [32, 80]. Similar to published reports, we found that after 30–36 days of differentiation, the AST- and the NAS-derived cells developed neuronal like morphology and expressed the markers of dopaminergic neurons such as tyrosine hydroxylase (TH) and dopamine transporter (DAT) [32, 80]. Importantly, almost all neuronal-like cells were silent, meaning they did not exhibit spontaneously firing activity, which is a hallmark of dopaminergic neuronal phenotype. After 150 days of differentiation, ~ 98% of human iPSC-derived neurons were observed to express all expected dopaminergic neuronal markers and importantly, these neurons were electrically mature with self-initiated firing activity, recapitulating the canonical dopaminergic neuronal phenotype. Via a double blinded experimental design, we report that α-synuclein overexpression, in both human-like iPSC-derived and primary mouse dopamine neurons, induces a unique firing pattern characterized by prolonged broadbrimmed bursts. Furthermore, we show that activation of D2R restores the firing pattern to baseline. Collectively our findings provide new insight into the pathophysiological events induced by α-synuclein and the mechanisms underlying the benefit of D2R agonists in Parkinson’s disease.

## Results

Inaccessibility of diseased tissue for mechanistic studies prior to neuronal demise creates major barrier to research on Parkinson's disease. Induced pluripotent stem cells (iPSCs) differentiated toward a dopaminergic neuronal phenotype offer a valuable source to generate human dopaminergic neurons. In this study, we differentiated two iPSC lines obtained from a patient with α-synuclein triplication (AST) and an unaffected first-degree relative (NAS), each of which were differentiated into dopamine neurons [32]. The overall goal of this study was to examine α-synuclein modulation of human dopamine neuronal activity prior to neuronal demise.

### Expression of human dopaminergic neuron markers and α-synuclein

**Fig. 1:**
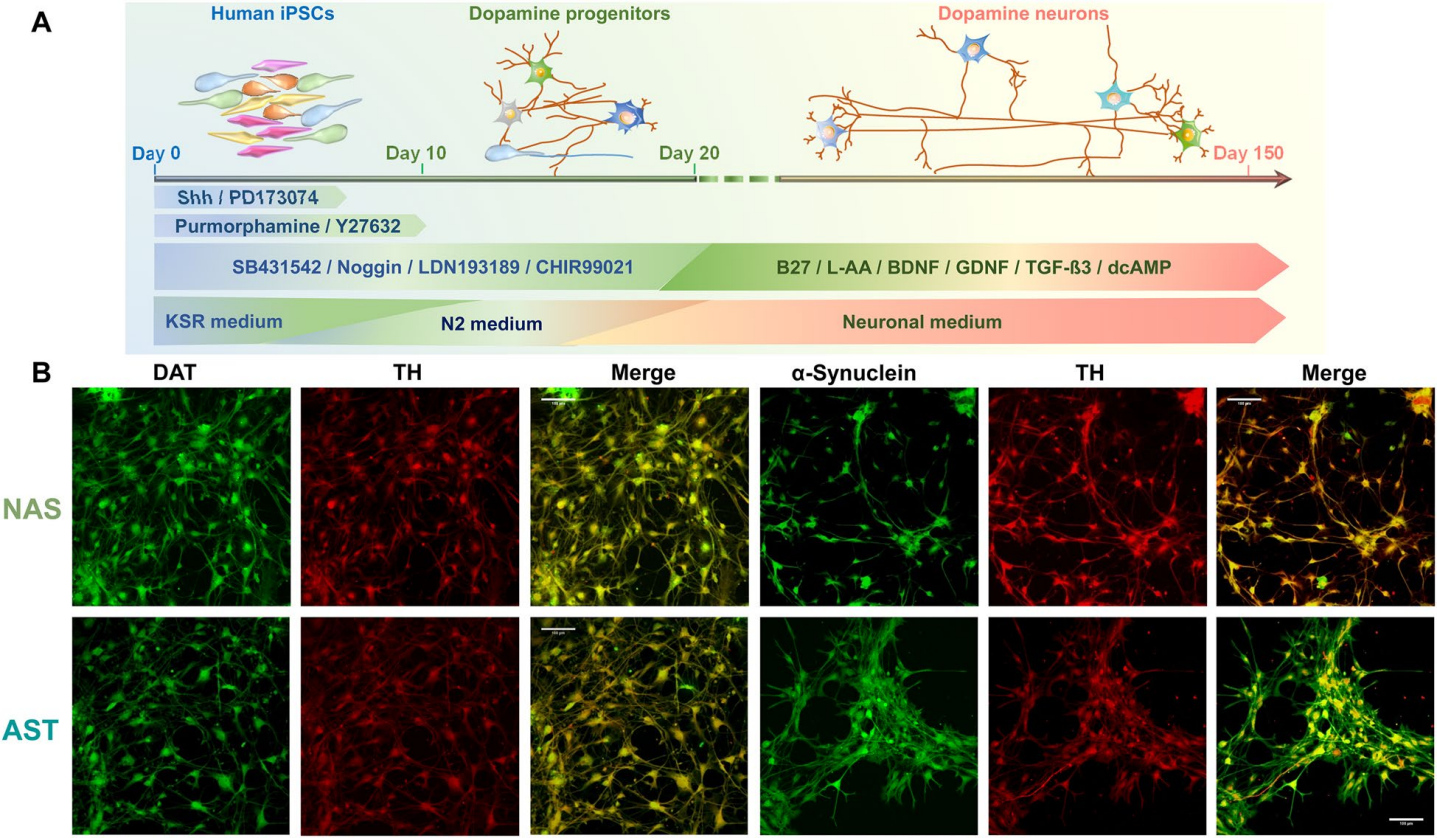
Overview of differentiation protocol and identification of the iPSCs-derived human-like dopaminergic neurons at early stage of differentiation. **a** Schematic depicts the timeline of differentiation protocol, growth factors/small molecules and culture medium used for differentiation. **b** Immunostaining for markers of dopaminergic neurons and α-synuclein after 36 days of differentiation. Cells were subject to immunostaining for two markers of dopaminergic neurons the dopamine transporter (DAT) and tyrosine hydroxylase (TH). Scale bar: 100 μm- All experiments and analyses are performed via a blinded experimental design

**Fig. 2:**
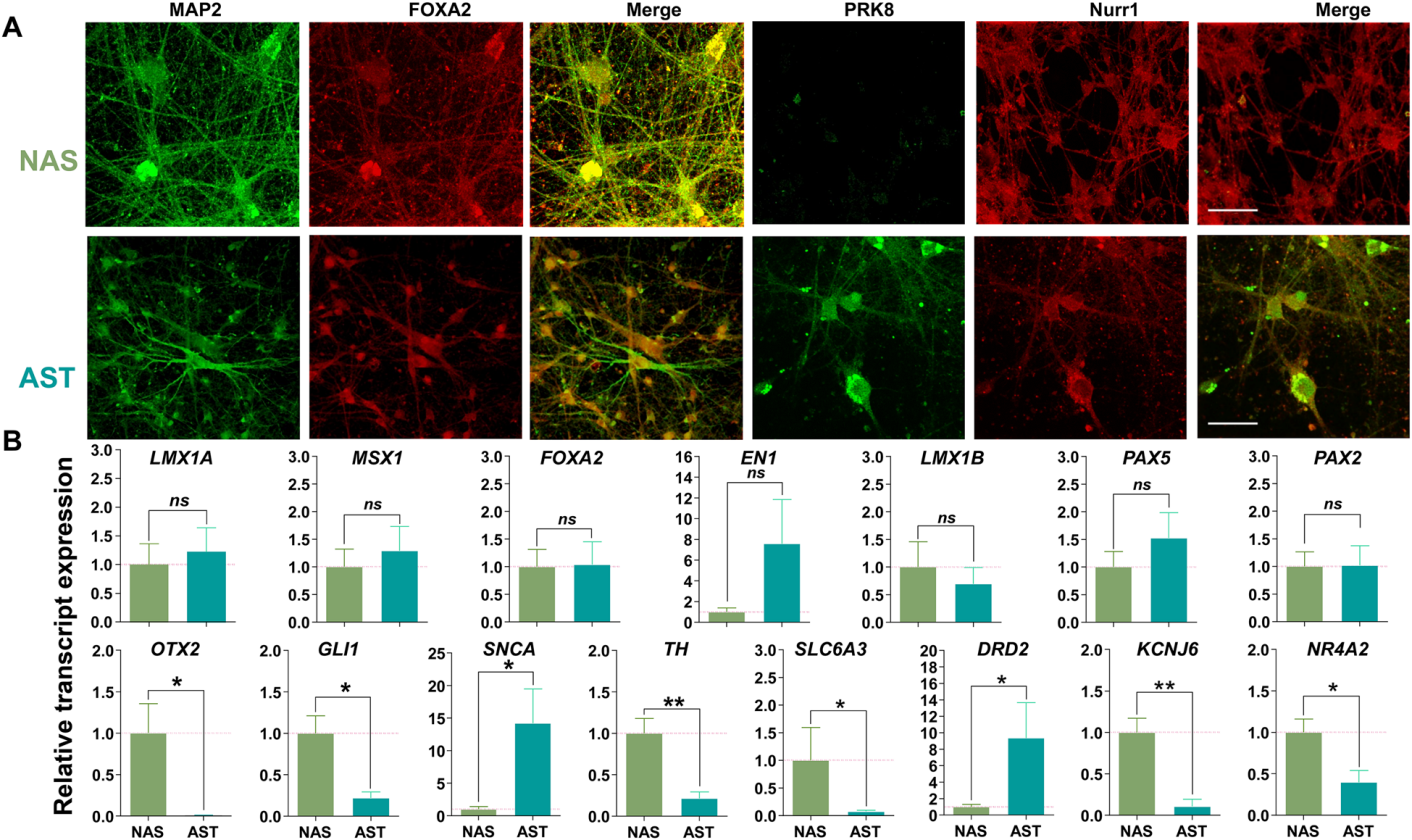
Identification of the midbrain dopamine neuron and Parkinson’ disease markers at late stages (5 months) of differentiation for NAS and AST cells. **a** After 5 months of differentiation, the immunostaining for MAP2, FOXA2, and Nurr1 show both NAS- and AST-derived dopaminergic neurons express these proteins. There is a noticeable increase in PRK8 immunostaining in AST-derived dopaminergic neurons. Scale bar: 100 μm. The immunostainings were performed in parallel via a blinded experimental design. **b** Histograms showing fold changes in the mRNA expression of selected genes in hiPSC-derived dopamine neurons with SNCA Triplication (AST, n = 6 cultures) compared to hiPSC-derived dopamine neurons with normal SNCA copy number (NAS, n = 6 cultures). GAPDH, 18S, and ACTB were chosen as housekeeping genes. Data expressed as means ± SEM and analyzed by an unpaired t-test. Differences in the data were considered significant if p < 0.05. The unpaired t-tests show no significant differences in LMX1A, MSX1, FOXA2, EN1, LMX1B, PAX2, and PAX5 mRNA expression between the two experimental groups, significant increases in SNCA and DRD2 mRNA expression in the AST group compared to the NAS group, and significant decreases in OTX2, GLI1, TH, SLC6A3, KCNJ6, and NR4A2 mRNA expression in the AST group compared to the NAS group. ns = not significant, * = significant when *p* < 0.05, and ** = significant when *p* < 0.01

As outlined in Fig. 1a, iPSCs were differentiated to dopaminergic neurons**.** To confirm that human iPSC-derived neurons expressed canonical dopaminergic neuronal markers, we used co-immunofluorescent labeling for either tyrosine hydroxylase (TH) and dopamine transporter (DAT), or co-labeling for α-synuclein and TH at an early differentiation stage (Fig. 1b). To identify whether α-synuclein overexpression affects the expression of dopaminergic neuronal markers, we utilized methodologies described in previous reports [21, 28, 78, 129] to quantitatively analyze the immunoreactivity of these markers. Quantifying average fluorescence intensity suggested increased immunoreactivity for α-synuclein in the AST-derived neurons and decreased immunoreactivity for DAT and TH (Additional file 1: Fig. S1). To verify complete neuronal differentiation, we surveyed the expression of canonical indicators for neuronal maturation such as Nurr1, FOXA2 and MAP2, followed by immunostaining for dopaminergic markers. The orphan nuclear receptor-related factor 1 (Nurr1) is involved in the development of midbrain dopamine neurons [137]. FOXA2 is a member of the Foxa subfamily of forkhead/winged helix transcription factors [3] and is required for the expression of Nurr1 in immature midbrain dopamine neurons and for the differentiation to mature midbrain dopamine neurons [36]. MAP2 provides information on cytoskeletal structure, specific to neurons [87, 15, 24, 31]. Genes encoding Parkin (PRK8, [64] and α-synuclein [100], have been linked to familial PD [113]. Therefore, we performed parallel immunostaining using identical solutions and imaging resolution to examine immunoreactivity for Nurr1, FOXA2, MAP2 and PRK8, (Fig. 2a). Consistent with previous data [32], we found all of these markers are expressed in both human-like dopamine neurons with normal α-synuclein levels (NAS) and α-synuclein triplication (AST). Notably, PRK8 is clearly detectable in AST-derived dopamine neurons, but only scattered punctate staining is detected in NAS-derived dopamine neurons (Fig. 2a). In addition, calculating the average fluorescence intensity for each marker suggested decreased immunoreactivity of MAP2, FOXA2, and Nurr1 (Additional file 1: Fig. S1).

The development of ventral midbrain dopamine neurons involves multiple parallel, controlled processes encompassing many transcription factors tightly coordinated in order to produce functional midbrain neurons [6]. Several factors have been identified and used to determine the fate of midbrain dopaminergic neurons in the embryonic brain, including Lmx1a [5], Msx1 [5], Foxa2 [36], En-1 [115], Lmx1b [117], Pax2/Pax5 [128], Otx2 [1], Gli1 [136], tyrosine hydroxylase [114], Slic6a3 [8], and Nurr1 [137]. We next investigated the transcript expression levels of these markers in the fully differentiated AST- and NAS-derived dopamine neurons using qPCR (Fig. 2B, n = 6 from three independent rounds of differentiation). Our results showed that the transcription factors outlined above were expressed in both NAS- and AST-derived dopamine neurons. Consistent with previous reports [98], [32], transcript levels for α-synuclein were increased in AST-derived dopamine neurons (n = 6 from three independent rounds of differentiation, two-tailed paired t-test, *p* = 0.04),whereas, the transcript levels for transcription factors OTX2 and GLI1 as well as for canonical markers TH, dopamine transporter, GIRK2 channels and Nurr1 were decreased (Fig. 2, n = 6 from three independent rounds of differentiation, two-tailed paired t-test, *p* = 0.0006). Notably, the transcript levels for D2 receptor were increased in the AST-derived dopamine neurons, but the mRNA levels for KCNJ6G, a gene encoding G-protein activated inward rectifier potassium channel 2 (GIRK2) were decreased (n = 6 from three independent rounds of differentiation, two-tailed paired t-test, *p* = 0.04). Notably, a shared limitation of qPCR and immunocytochemistry is that one cannot ascertain the membrane levels or the activity of these membrane-embedded receptors. Nevertheless, collectively, these data suggest that our differentiation protocol produces human iPSC-derived dopaminergic neurons expressing their appropriate markers. Although the results shown in this study have been reproducible in multiple rounds of differentiation, one of the limitations of this study is that they are conducted in two iPSC lines, normal and α-synuclein triplication lines.

### α-synuclein-overexpressing dopamine neurons exhibit a unique pattern of spontaneous firing activity with a pause between subsequent broadbrimmed bursts

To further elucidate the pathophysiological effects of α-synuclein on dopamine neurons, we next examined the intrinsic firing behavior of NAS- and AST-derived dopamine neurons after 5 months of neuronal differentiation. A total of 157 iPSC-derived dopamine neurons from three independent rounds of differentiation were recorded and analyzed for the experiments outlined below. Experiments and analyses were performed under a blinded experimental design. The primary parameters of passive membrane were averaged for each NAS and AST group. The average input resistance was 312.4 ± 58.4 MΩ in NAS and 135.7 ± 16.6 MΩ in AST (*p* = 0.003, two-tailed Student's t tests); the membrane time constant was 897.8 ± 61.7 μs in NAS and 918.2 ± 54.7 in AST (*p* = 0.802, two-tailed Student's t tests); and the membrane capacitance was 66.4 ± 4.5 pF in NAS and 88.4 ± 6.0 pF in AST (*p* = 0.005, two-tailed Student's t tests).

**Fig. 3:**
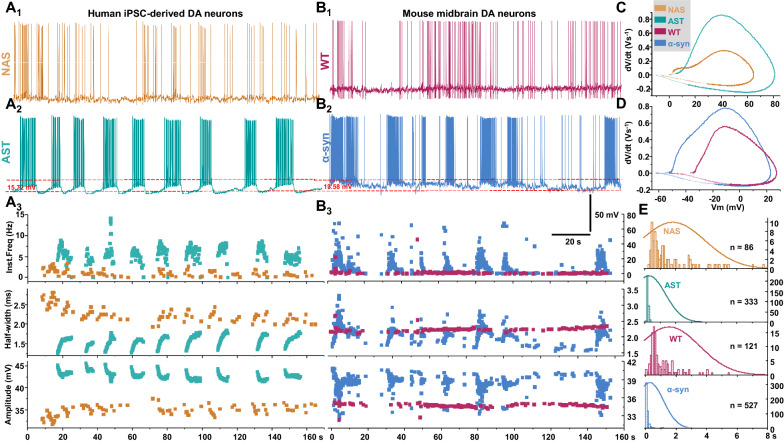
Both AST-derived dopamine neurons and mouse dopamine neurons overexpressing α-synuclein exhibit bursting firing pattern. The experiments and analyses were performed in parallel and via a blinded experimental design. **a**_**1**_ Representative trace of a spontaneously active NAS-derived dopamine neurons shows a mixture of single spikes and small burst activity with an underlying “pacemaker-like” periodicity. **a**_**2**_ Representative trace of a spontaneously active AST-derived dopamine neuron shows a unique high up-state (depolarized plateau) and high frequency of spontaneous activity with a pause between subsequent broadbrimmed bursts. **a**_**3**_ Time courses of instantaneous firing frequency, action potential half-width and amplitude obtained from (a_1_) and (a_2_). **b**_**1**_ Representative trace of a spontaneously active wild type mouse dopamine neuron obtained from midbrain primary neuronal culture. **b**_**2**_ Representative trace of a spontaneously active mouse dopamine neuron overexpressing α-synuclein. **b**_**3**_ Time courses of instantaneous firing frequency, action potential half-width and amplitude obtained from b_1_ and b_2_. **c** Phase-plane plot of action potentials generated from (a_1_) and (a_2_). Unlike AST-derived dopamine neurons, the action potentials in NAS-derived dopamine neurons show a smooth and slower onset at initiation site. **d** Phase-plane plot of action potentials generated from b_1_ and b_2_. **e** Interspike histogram (in ms) from top to bottom: NAS-, AST-derived neurons, WT mouse dopamine neurons, and mouse dopamine neurons overexpressing α-synuclein. n corresponds to the total number of spikes for each histogram

Previous studies report on the firing behavior of wild type (WT) rodent dopamine neurons [12, 46, 48, 52, 55, 75, 76, 84, 108, 110, 124] and have shown WT dopamine neurons exhibit both pacemaker-like firing activity and burst firing with an average rate of 0.5–10 Hz [46]. Studies also demonstrate a broader range of spontaneous spike frequency of 0–20 Hz in dopamine neurons [66]. In this study, we found the majority of NAS-derived dopamine neurons exhibited a mixture of single spikes and small burst activity with an underlying “pacemaker-like” periodicity. The pacemaker-like firing activity and canonical irregular single-spike firing occurred at rates of 1–4 Hz. Bursts of 3–8 spikes occurred at higher frequencies with a pause between subsequent bursts with a varied interspike interval (Fig. 3a1), whereas the majority of AST-derived dopamine neurons (~ 90% i.e., 25 of 28 recorded neurons) exhibited a unique high up-state (depolarized plateau) and high frequency of spontaneous activity with a pause between subsequent broadbrimmed bursts (Fig. 3a2). The burst range was 5–25 Hz (average: 11.6 ± 1.4 Hz) with 30–300 spikes (118.1 ± 19.8 spikes) in 5–25 s duration (11.2 ± 1.5 s, n = 27/group).

To determine whether increased α-synuclein in AST-derived dopamine neurons is the underlying mechanism for the observed firing behaviors, we measured the firing activity of WT and α-synuclein-overexpressing mouse midbrain dopamine neuronal culture. Consistent with previous reports [12, 13, 52, 75, 76, 108, 110], the firing pattern of cultured mouse dopamine neurons is a mixture of single spikes and small burst activity with an underlying “pacemaker-like” periodicity that is similar to the firing activity of NAS-derived dopamine neurons (Fig. 3b1). Similar to the firing pattern measured in the AST-derived dopamine neuron, in mouse dopamine neurons overexpressing α-synuclein, the frequency of spontaneous firing activity is increased (Fig. 3a3, b3), coupled with a depolarized plateau (high up-state, Fig. 3a2, b2) and a pause between broadbrimmed bursts (Fig. 3b2).

The phase plot is generated when change in the time derivative of the voltage (dV/dt) is plotted against the membrane potential V(t) [58, 91]. Examining the phase plot shapes for the action potential upstroke (Fig. 3c, d) revealed the rate of membrane potential change in the AST-derived dopamine neurons (Fig. 3c) is similar to α-synuclein-overexpressing mouse dopamine neurons (Fig. 3d). Additionally, the action potential onsets in NAS-derived dopamine neurons (Fig. 3c) and WT mouse dopamine neurons (Fig. 3d) are relatively slower at the initiation site. The interspike interval (ISI) distribution is a commonly used criterion to determine if burst activity is present in single neurons [83, 91]. The ISI of an NAS-derived dopamine neuron and dopamine neuron from a WT mouse are randomly distributed within 0.2–6 s (Fig. 3e). Conversely, the range of ISI distribution in an AST-derived dopamine neuron and an α-synuclein-overexpressing mouse dopamine neuron fall in a narrower ranged below 1 s (Fig. 3e). Overall, these data indicate that increases in α-synuclein alter the pattern of intrinsic firing activity in both human iPSC-derived dopamine neurons and mouse dopamine neurons.

### Increased α-synuclein levels underly the altered intrinsic firing behavior of AST-derived dopamine neurons

**Fig. 4:**
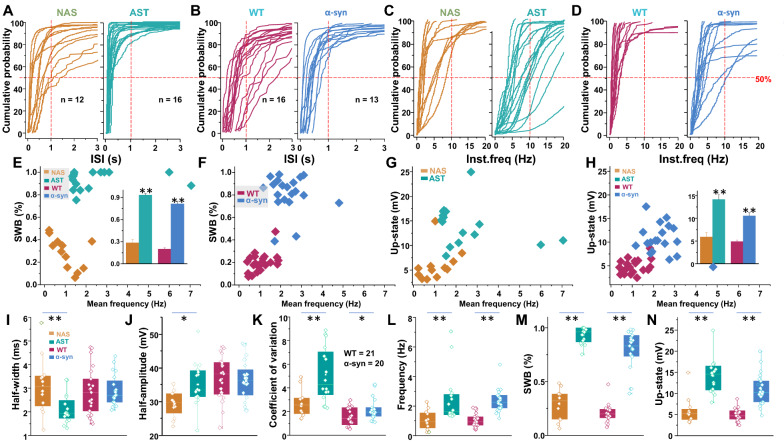
Analyses of firing-pattern and intrinsic firing properties of NAS-, AST-derived neurons, WT mouse dopamine neurons, and mouse dopamine neurons overexpressing α-synuclein. The experiments and analyses were performed in parallel via a blinded experimental design. **a** left: Interspike interval (ISI) distribution in NAS-derived dopamine neurons. Each curve in individual panels represents cumulative probability for an individual neuron, the 50% probability was below 1 s. Right: ISI distribution in AST-derived dopamine neurons, the 50% probability was below 0.25 s. **b** Left: ISI distribution of WT mouse dopamine neurons, the 50% probability was below 2 s. Right: ISI distribution observed in mouse dopamine neurons overexpressing α-synuclein. The 50% probability was below 0.5 s. **c** Left: Instantaneous frequency distribution obtained from NAS-derived neurons; the 50% probability was below 10 Hz. Right: Instantaneous frequency distribution obtained from AST-derived neurons; the 50% probability was below 20 Hz. **d** Left: Instantaneous frequency distribution of WT mouse dopamine neurons, the 50% probability was below 5 Hz. Right: Instantaneous frequency distribution of mouse dopamine neurons overexpressing α-synuclein, the 50% probability was below 10 Hz. **e** Mean firing frequency (Hz) against percentage of spike within a burst (%SWB) for NAS- and AST-derived dopamine neuron groups. Inset shows increased α-synuclein in the neurons enhanced %SWB in both iPSC-derived human-like dopamine neurons and mouse dopamine neurons. **f** Mean firing frequency against %SWB for WT mouse dopamine neurons, and mouse overexpressing α-synuclein. **g** and** h** Mean firing frequency against up-state for NAS- and AST-derived dopamine neurons, or WT mouse dopamine neurons against mouse dopamine neurons overexpressing α-synuclein. Inset shows a higher amplitude of up-state in both AST-derived dopamine neurons and mouse dopamine neurons overexpressing α-synuclein. **I**. The spike half-width of AST-derived dopamine neurons (n = 16) is shorter than NAS-derived neurons (n = 12, F_(3, 65)_ = 3.744, *p* = 0.015, one-way ANOVA). **j** The spike half-amplitude of AST-derived neurons is higher than NAS-derived neurons (F_(3, 65)_ = 4.681, *p* = 0.005, one-way ANOVA). **k** interspike intervals are calculated over 1 min of spontaneous firing activity, the coefficients of variation of the interspike interval are significantly larger in either AST-derived dopamine neurons or mouse dopamine neurons overexpressing α-synuclein compared to NAS-derived dopamine neurons or WT mouse dopamine neurons (F_(3, 63)_ = 21.39, *P* < 0.0001. one-way ANOVA). **l** Compared to NAS-derived dopamine neurons or WT mouse dopamine neurons, the spontaneous firing rates are higher in AST-derived dopamine neurons or mouse dopamine neurons overexpressing α-synuclein (F_(3, 65)_ = 10.67, *P* < 0.0001, one-way ANOVA). **m** Compared to their counterpart experimental group, the percentage of spike within a burst is higher in AST-derived dopamine neurons or mouse dopamine neurons overexpressing α-synuclein (F_(3, 67)_ = 160.8, *P* < 0.0001, one-way ANOVA). **n** The up-state is significantly higher in either AST-derived dopamine neurons or mouse dopamine neurons overexpressing α-synuclein, compared to their counterpart experimental groups containing endogenous α-synuclein level (F_(3, 67)_ = 34.57, *P* < 0.0001, one-way ANOVA)

To investigate the underlying mechanism of altered intrinsic firing behavior of dopamine neurons induced by excess α-synuclein levels, we compared the distribution of individual ISI in the AST-derived versus NAS-derived neurons as well as in α-synuclein-overexpressing mouse dopamine neurons versus WT dopamine neurons (Fig. 4a–d). The 50% probability of ISI in AST-derived dopamine neurons (Fig. 4a, n = 16) and α-synuclein-overexpressing dopamine neurons (Fig. 4b, n = 13) were below 0.5 s. In contrast, the 50% probability of ISI in NAS-derived neurons and WT dopamine neurons were over 1 s. Accordingly, analysis of individual dopamine neuron for firing frequency exhibited the 50% probability of instantaneous frequency were below 10 Hz in NAS-derived neurons and below 5 Hz in WT dopamine neurons. In comparison, the 50% probability of instantaneous frequency were over 10 Hz in both AST-derived dopamine neuron and mouse dopamine neurons overexpressing α-synuclein (Fig. 4c, d).

To elucidate alterations in patterns of firing activity beyond average firing rate, we evaluated firing activity for bursting features, defined as periods of high frequency firing of a neuron separated by periods of quiescence, which has been observed in various neuronal systems, both in vitro and in vivo [25, 46, 82]. A wide variety of computational approaches have been developed to detect periods of bursting in spike trains. We used the algorithm developed by Grace and Bunney [46] to compare bursting in dopamine neurons with native vs. increased α-synuclein levels. Burst onsets were defined by two consecutive spikes with an ISI < 80 ms and terminated when the ISI was > 160 ms. Spikes that occurred within the burst are expressed relative to the total number of spikes from the same neuron (%SWB) [46]. Burst analysis revealed that 28.5 ± 4.2% of spikes in NAS-derived dopamine neurons were within bursts. In striking contrast, 93.2 ± 2% of spikes in the AST-derived neurons were within a burst (%SWB, Fig. 4e, m). Similarly, mouse dopamine neurons overexpressing α-synuclein produced a much higher percentage of spikes fired in bursts (%SWB, 81.2 ± 3.7%, range of 40% to 97% in individual neurons) compared to WT dopamine neurons (%SWB, 20.0 ± 1.9%, range of 8% to 47% in individual neurons) (Fig. 4f, m). Further examination of membrane potential during periods of quiescence and bursting revealed significant relative changes in up-state potential. Up-state refers to the membrane potential during a firing burst, which is depolarized relative to quiescent periods, known as down-states [73]. The up-state was increased in both AST-derived neurons and mouse dopamine neurons overexpressing α-synuclein (NAS = 5.91 ± 1.0 mV vs. AST = 14.13 ± 1.1 mV, *p* < 0.001,WT mouse = 4.88 ± 0.3 mV vs. mouse dopamine neurons overexpressing α-synuclein = 10.54 ± 0.7 mV, *p* < 0.001, Fig. 4g, h, n). Increased up-state was coupled to a higher firing frequency (NAS = 1.04 ± 0.2 Hz vs. AST = 2.46 ± 0.4 Hz, *p* = 0.011,WT mouse = 1.04 ± 0.2 Hz vs. mouse dopamine neurons overexpressing α-synuclein = 2.36 ± 0.2 Hz, *p* < 0.001, Fig. 4l), with a larger coefficient-of-variation (NAS = 2.70 ± 0.3 vs. AST = 5.11 ± 0.6, *p* = 0.002,WT mouse = 1.58 ± 0.2 vs. mouse dopamine neurons overexpressing α-synuclein = 2.19 ± 0.2, *p* = 0.03, Fig. 4k). Additionally, the up-state in AST-derived dopamine neurons (Fig. 4g, n) or mouse dopamine neurons overexpressing α-synuclein (Fig. 4h, n) revealed higher frequencies of distribution. Finally, examination of action potential morphology showed the AST-derived dopamine neurons exhibited a significantly shorter half-width of action potential (2.12 ± 0.16 ms, Fig. 4I) and higher amplitude (35.17 ± 1.7 mV Fig. 4j) compared to NAS-derived dopamine neurons (half-width: 3.11 ± 0.36 ms, *p* = 0.015, half-amplitude: 29.3 ± 1.16 mV, one-way ANOVA, *p* = 0.005, Fig. 4i, j). Collectively, these data suggest increased α-synuclein levels modulates the intrinsic firing behavior of both AST-derived human like dopamine neurons and mouse dopamine neurons overexpressing α-synuclein.

### Activation of dopamine D2 receptors reinstates the firing activity of AST- to NAS-derived dopamine neurons levels

**Fig. 5:**
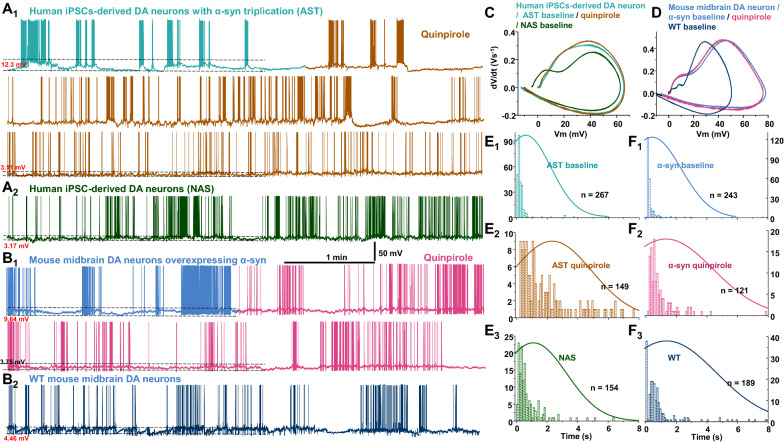
Agonist activation of D2R reduces the size of clustered burst firing in AST-derived dopamine neurons or mouse dopamine neurons overexpressing α-synuclein. The experiments and analyses were performed in parallel and via a blinded experimental design. **a**_**1**_ Representative recording of a spontaneously active AST-derived dopamine neuron before (aqua blue) and during (brown) application of quinpirole. **a**_**2**_ Representative trace of a spontaneously active NAS-derived dopamine neurons shows a mixture of single spikes and burst activity. **b**_**1**_ Representative recording of a spontaneously active mouse dopamine neuron overexpressing α-synuclein before (navy-blue) and during (wine) application of quinpirole. **b**_**2**_ Representative trace of a spontaneously active wild type mouse dopamine neuron obtained from midbrain primary neuronal culture. **c** Phase-plane plot of action potentials generated from (**a**_**1**_) and (**a**_**2**_). **d** Phase-plane plot of action potentials generated from (**b**_**1**_) and (**b**_**2**_) **e** Interspike histogram (in ms) of human iPSCs-derived dopamine neurons for three conditions belonging, from top to bottom: AST-derived neurons at baseline (**e**_**1**_), during quinpirole application (**e**_**2**_) and NAS baseline (**e**_**3**_). **f** Interspike histogram (in ms) of mouse midbrain dopamine neurons for three conditions belonging, from top to bottom: overexpressing α-synuclein at baseline (**f**_**1**_), during quinpirole application (**f**_**2**_) and WT baseline (**f**_**3**_). n corresponds to the total number of spikes to determine the histogram

Previous studies have shown that activation or inhibition of dopamine D2 receptors (D2Rs) tightly regulates the firing activity of dopamine neurons [11, 19, 23, 37, 56, 90, 96]. Decreased functionality or inhibition of D2 receptors produces a distinct firing pattern that is described by increased up-state leading to a higher firing frequency of dopamine neurons, which is similar to what we measured in the AST-derived dopamine neurons and mouse dopamine neurons overexpressing α-synuclein. Therefore, we reasoned that the distinct firing pattern observed in these neurons (Figs. 3, 4) might be due to the decreased functional availability of D2R. To test this possibility, we activated D2 receptors on AST-derived dopamine neurons (Fig. 5a1) or mouse dopamine neurons overexpressing α-synuclein (Fig. 5a2) using the D2R agonist quinpirole (5 μM). Bath application of quinpirole dispersed broadbrimmed firing burst into NAS-derived or WT mouse dopamine neurons-like, smaller bursts intermingled single spikes (brown trace for AST-derived neurons, wine trace for mouse neurons overexpressing α-synuclein). Although quinpirole did not affect the action potential onset (Fig. 5c, d), the ISI distribution was restored to the time window measured in NAS-derived (Fig. 5e3) and WT dopamine neuron levels (Fig. 5f3).

**Fig. 6:**
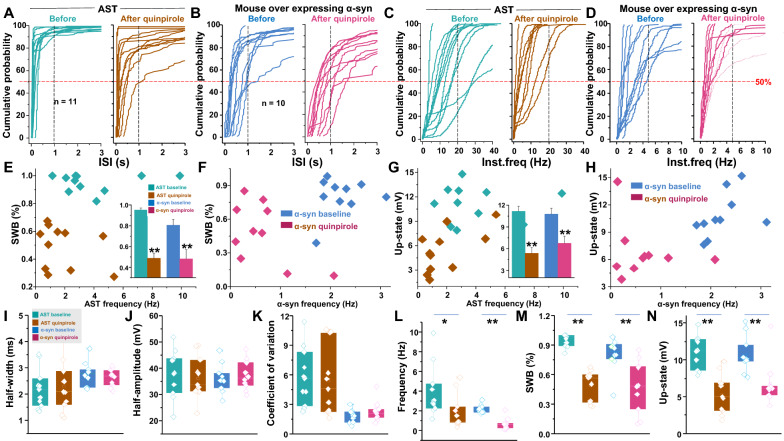
Agonist activation of D2R modulated the intrinsic firing properties of AST-derived dopamine neurons and mouse dopamine neurons overexpressing α-synuclein. In these neurons, bath application of quinpirole dispersed broadbrimmed burst firing to mixture of single spikes and small burst activity with an underlying “pacemaker-like” periodicity. **a** Left: interspike interval (ISI) distribution in AST-derived dopamine neurons. The 50% probability was below 0.25 s. Right: ISI distribution observed in AST-derived dopamine neurons after quinpirole application, the 50% probability was below 1 s. **b** Left: ISI distribution in mouse dopamine neurons overexpressing α-synuclein. Right: ISI distribution observed during quinpirole applications in mouse dopamine neurons overexpressing α-synuclein. **c** Left: Instantaneous frequency distribution in AST-derived dopamine neurons. Right: Instantaneous frequency distribution obtained from AST-derived dopamine neurons during quinpirole applications. **d** Left: instantaneous frequency distribution in mouse dopamine neurons overexpressing α-synuclein. Right: Instantaneous frequency distribution during quinpirole applications in mouse dopamine neurons overexpressing α-synuclein. **e** Mean firing frequency is plotted against %SWB of AST-derived DA neurons before (aqua blue) and during quinpirole applications (brown). Inset shows quinpirole reduces %SWB in both experimental groups. **f** Mean firing frequency is plotted against %SWB in mouse dopamine neurons overexpressing α-synuclein before (navy-blue) and during quinpirole applications (wine). **g** Mean firing frequency is plotted against up-state for AST-derived dopamine neurons before (aqua blue) and during quinpirole applications (brown). Inset shows quinpirole reduces the amplitude of up-state in both experimental groups. **h** Mean firing frequency is plotted against up-state for mouse dopamine neurons overexpressing α-synuclein before (navy-blue) and during quinpirole applications (wine). **i** and **j** Quinpirole application does not change the spike half-width or spike half-amplitude in either AST-derived dopamine neurons or mouse dopamine neurons overexpressing α-synuclein. **k** Interspike intervals are calculated over 1 min of spontaneous firing activity. Quinpirole application does not change the coefficients of variation of the interspike intervals in either AST-derived dopamine neurons or mouse dopamine neurons overexpressing α-synuclein. **l**, **m** and **n** Quinpirole decreases the spontaneous firing rates (**l**, baseline: 3.9 ± 0.8 Hz; quinpirole: 1.8 ± 0.5 Hz, n = 11, *p* < 0.05), %SWB (**m**, baseline: 95.1 ± 2%; quinpirole: 48.8 ± 4.9%, n = 11, *p* < 0.01) and up-state (**n**, baseline: 11.2 ± 0.7 mV; quinpirole: 5.4 ± 0.8 mV, n = 11, *p* < 0.01) in AST-derived dopamine neurons and in mouse dopamine neurons overexpressing α-synuclein (firing rates: baseline: 2.2 ± 0.1 Hz, quinpirole: 0.6 ± 0.2 Hz, *p* < 0.01; %SWB: baseline: 80.6 ± 5.5%; quinpirole: 48.2 ± 8.9% n = 12, *p* < 0.01; up-state: baseline: 10.8 ± 0.8 mV, quinpirole: 6.7 ± 1.0 mV, n = 12, *p* < 0.01). The experiments and analyses were performed in parallel and via a blinded experimental design

We investigated the distribution of individual ISI in AST-derived dopamine neurons and mouse dopamine neurons overexpressing α-synuclein (Fig. 6a, b) before and during bath application of quinpirole (5 μM). Quinpirole shifted the 50% cumulative probability of ISI from low to high values in both AST-derived dopamine neurons (Fig. 6a) and mouse dopamine neurons overexpressing α-synuclein (Fig. 6b). Similarly, quinpirole decreased the 50% cumulative probability of instantaneous firing frequency (Fig. 6c, d). In addition, quinpirole decreased the %SWB, average firing frequency, and the magnitude of up-state in both AST-derived dopamine neurons and mouse dopamine neurons overexpressing α-synuclein (Fig. 6i–n). Specifically, in AST-derived dopamine neurons %SWB decreased from 95.1 ± 2% to 48.8 ± 4.9% (*p* < 0.001, n = 11, Fig. 6e inset, m). In mouse dopamine neurons overexpressing α-synuclein, %SWB decreased from 80.6 ± 5.5% to 48.2 ± 8.9% (*p* = 0.004, n = 12) (Fig. 6f, m). Quinpirole reduced the firing frequency from 3.9 ± 0.8 Hz to 1.8 ± 0.5 Hz in AST-derived dopamine neurons and from 2.2 ± 0.1 Hz to 0.6 ± 0.2 Hz in mouse dopamine neurons overexpressing α-synuclein (Fig. 6l). Quinpirole suppressed the up-state amplitude in both AST-derived dopamine neurons (from 11.2 ± 0.7 mV to 5.4 ± 0.8 mV, *p* < 0.001, n = 11, Fig. 6g inset) and in mouse dopamine neurons overexpressing α-synuclein (from 10.8 ± 0.8 mV to 6.7 ± 1.0 mV, *p* = 0.003, n = 12, Fig. 6h, n). Notably, quinpirole did not affect action potential width, amplitude or coefficient of variation of firing frequency in either AST-derived dopamine neurons or mouse dopamine neurons overexpressing α-synuclein. Taken together, these results support the interpretation that while increases in α-synuclein in human-like or mouse dopamine neurons alter neuronal firing behavior, agonist activation of D2R reinstates innate neuronal activity. Furthermore, these data suggest that D2R functional availability may be decreased at baseline in dopamine neurons overexpressing α-synuclein.

### Dopamine neurons overexpressing α-synuclein exhibited low sensitivity to inhibition of dopamine D2 receptors

**Fig. 7:**
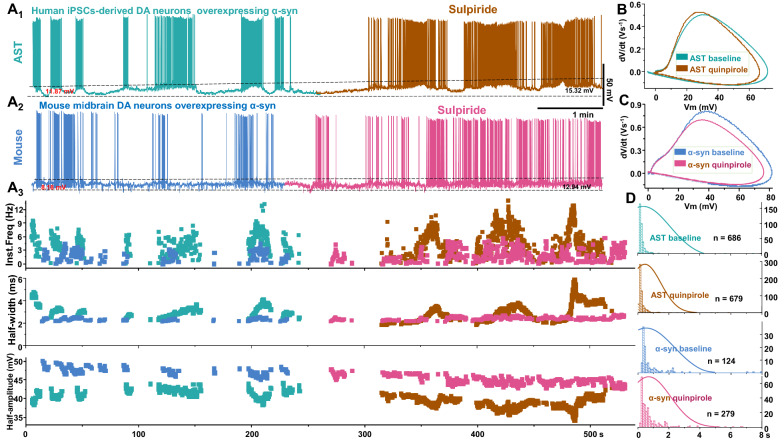
Blockade of dopamine D2R did not changed the firing frequency and ISI distributions in AST-derived-neurons or mouse dopamine neuron overexpressing α-synuclein. The experiments and analyses were performed via a blinded experimental design. **a**_**1**_ A representative recording of a spontaneously firing AST-derived dopamine neuron before (aqua blue) and after (brown) application of sulpiride (5 μM). **a**_**2**_ A Representative recording of a spontaneously firing mouse dopamine neuron overexpressing α-synuclein before (navy-blue) and after (wine) application of sulpiride (5 μM). **a**_**3**_ Time courses of instantaneous firing frequency, action potential half-width and amplitude obtained from (a_1_) and (a_2_). **b** Phase-plane plot of action potentials generated from (a_1_). **c** Phase-plane plot of action potentials generated from (a_2_). **d** Interspike histogram (in ms) for four conditions, from top to bottom: baseline and during sulpiride application for AST-derived dopamine, baseline and during sulpiride application for mouse dopamine neurons overexpressing α-synuclein. n corresponds to the total number of spikes used to determine the histogram

Next, we reasoned if the functional availability of D2R is limited in the AST-derived dopamine neurons and in mouse dopamine neurons overexpressing α-synuclein, then antagonism of D2R would produce little to no effect on their intrinsic firing behaviors included firing frequency, the %SWB and ISI distributions. As expected, although sulpiride, a D2R antagonist, mildly increased up-state and the size of broadbrimmed firing burst in both of AST (Fig. 7a1 brown) and mouse dopamine neuron overexpressing α-synuclein (Fig. 7a2 wine), there was no difference in firing frequency, half-width, half-amplitude (Fig. 7a3, b3), phase plot (Fig. 7b, c) and ISI distribution (Fig. 7d).

**Fig. 8:**
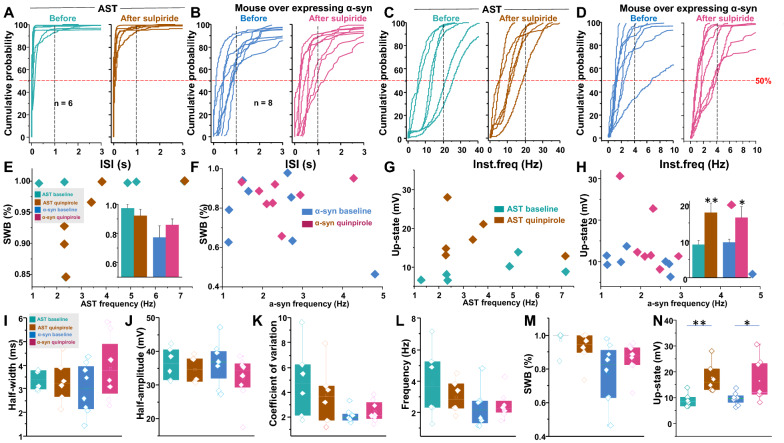
Blockade of D2R did not affected the intrinsic firing behaviors in the AST-derive dopamine neurons, or mouse dopamine neuron overexpressing α-synuclein. **a** Left: interspike interval (ISI) distribution observed in AST-derived dopamine neurons. The 50% probability was below 0.25 s. Right: ISI distribution observed in AST-derived dopamine neurons after sulpiride applications (5 μM), the 50% probability was below 0.25 s. **b** Left: ISI distribution observed in mouse dopamine neurons overexpressing α-synuclein. Right: ISI distribution observed in mouse dopamine neurons overexpressing α-synuclein during sulpiride applications (5 μM). **c** Left: Instantaneous frequency distribution obtained from AST-derived dopamine neurons. Right: Instantaneous frequency distribution obtained from AST-derived dopamine neurons during sulpiride applications. **d** Left: Instantaneous frequency distribution obtained from mouse dopamine neurons overexpressing α-synuclein. Right: Instantaneous frequency distribution obtained from mouse dopamine neurons overexpressing α-synuclein during sulpiride applications. **e** Mean firing frequency is plotted against %SWB for the AST-derived dopamine neurons before (aqua blue) and during sulpiride applications (brown). Inset shows sulpiride did not affect %SWB in either AST-derived dopamine neurons or mouse dopamine neurons overexpressing α-synuclein. **f** Mean firing frequency plotted against %SWB for mouse dopamine neurons overexpressing α-synuclein before (navy-blue) and during sulpiride applications (wine). **g** Mean firing frequency is plotted against up-state for the AST-derived dopamine neurons before (aqua blue) and during sulpiride applications (brown). **h** Mean firing frequency is plotted against up-state for mouse dopamine neurons overexpressing α-synuclein before (navy-blue) and during sulpiride applications (wine). Inset shows sulpiride significantly increased up-state in both AST-derived dopamine neurons and mouse dopamine neurons overexpressing α-synuclein. **i** and **j** Sulpiride did not affect the spike half-width the spike half-amplitude in either experimental groups (AST-derived dopamine neurons or mouse dopamine neurons overexpressing α-synuclein). **k**, **l**, **m**, and **n** Sulpiride application did not produce additional change in: coefficient of variation of the interspike intervals (**k,** *p* > 0.05; one-way ANOVA followed by Tukey's test, n = 6 for AST, n = 8 for mouse), spontaneous firing rates (**l,** *p* > 0.05; one-way ANOVA followed by Tukey's test, n = 6 for AST, n = 8 for mouse), and %SWB (**m**, *p* > 0.05; one-way ANOVA followed by Tukey's test, n = 6 for AST, n = 8 for mouse), but increased up-state (**n,** *p* < 0.05; one-way ANOVA followed by Tukey's test, n = 6 for AST, n = 8 for mouse) in either experimental groups

Contrary to the effect of D2R agonist, inhibition of D2R had no effect on the distribution of individual ISI or instantaneous frequency in either AST-derived dopamine neurons (Fig. 8a for ISI, 8c for instantaneous frequency) or mouse dopamine neurons overexpressing α-synuclein (Fig. 8B for ISI, 8d for instantaneous frequency). No group differences were observed in either firing frequency (Fig. 8l) and %SWB (Fig. 8e, m) after sulpiride treatment, even though it mildly increased the magnitude of the up-state in both of AST-derived dopamine neurons (Fig. 8g, n) and mouse dopamine neurons overexpressing α-synuclein (Fig. 8h, n). Moreover, inhibition of D2R did not produce additional reduction in the half-width (Fig. 8i), decrease the half-amplitude (Fig. 8j), or increase in the coefficient of variation of the ISI in the AST-derived or mouse dopamine neurons overexpressing α-synuclein (Fig. 8k). These data suggest that α-synuclein decreases the functional availability of D2R in the AST-derived dopamine neurons and mouse dopamine neurons overexpressing α-synuclein.

### NAS- and AST-derived dopamine neurons express D2 receptor and GIRK channels known to regulate their firing activity

In addition to the canonical markers of dopamine transporter and tyrosine hydroxylase, dopamine neurons express presynaptic D2 receptors and G protein-coupled inwardly rectifying potassium channel (GIRK) that self-regulate dopamine neuron activity [40, 59]. Importantly, GIRK2 expression within dopamine neurons is widely believed to distinguish the substantia nigra type dopamine neuron from the ventral tegmental area type dopamine neuron [38, 54, 67, 89, 125, 132]. Since our data suggest that D2R activation rescues α-synuclein-induced dysregulation of firing activity, we tested whether or not direct activation of GIRK would produce the same effect. Bath application of ML297 (10 μM), a GIRK channel activator, decreased the size of bursts, increased the ISI, and suppressed the upstate of AST-derived dopamine neurons, but only co-application of ML297 and quinpirole converted the AST firing behavior back to NAS-like firing (Additional file 1: Fig. S3). Furthermore, inhibition of GIRK channels or combined inhibition of GIRK channels and D2Rs worsens the increased up-state and firing frequency in the AST-derived dopamine neurons (Additional file 1: Fig. S4). Taken together, these data support the hypothesis that the D2R-GIRK pathway is disrupted and contributes to the abnormal firing activity of AST-derived dopamine neurons.

**Fig. 9:**
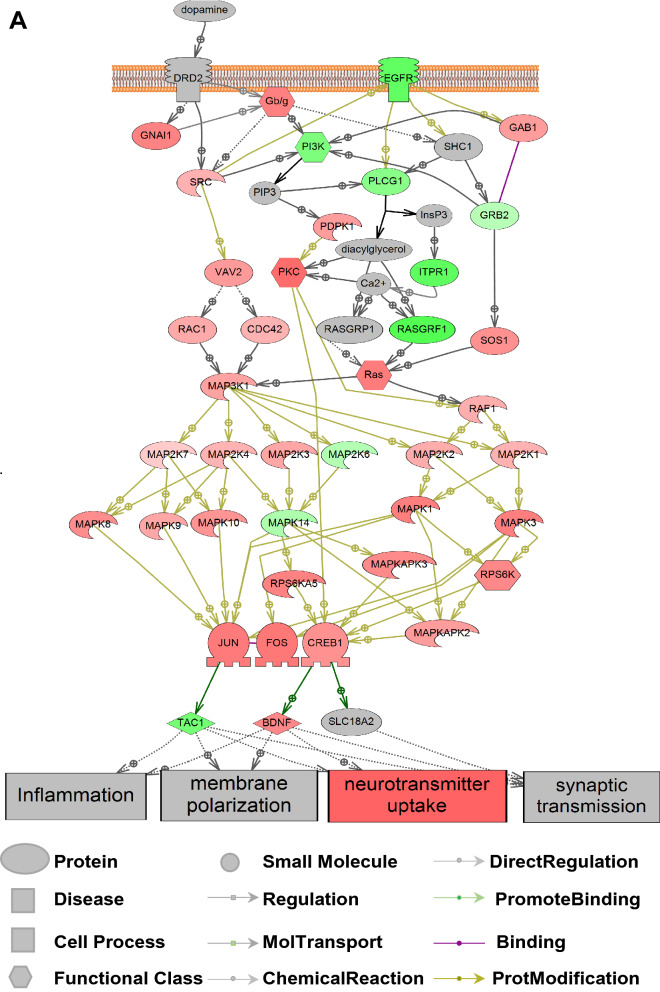

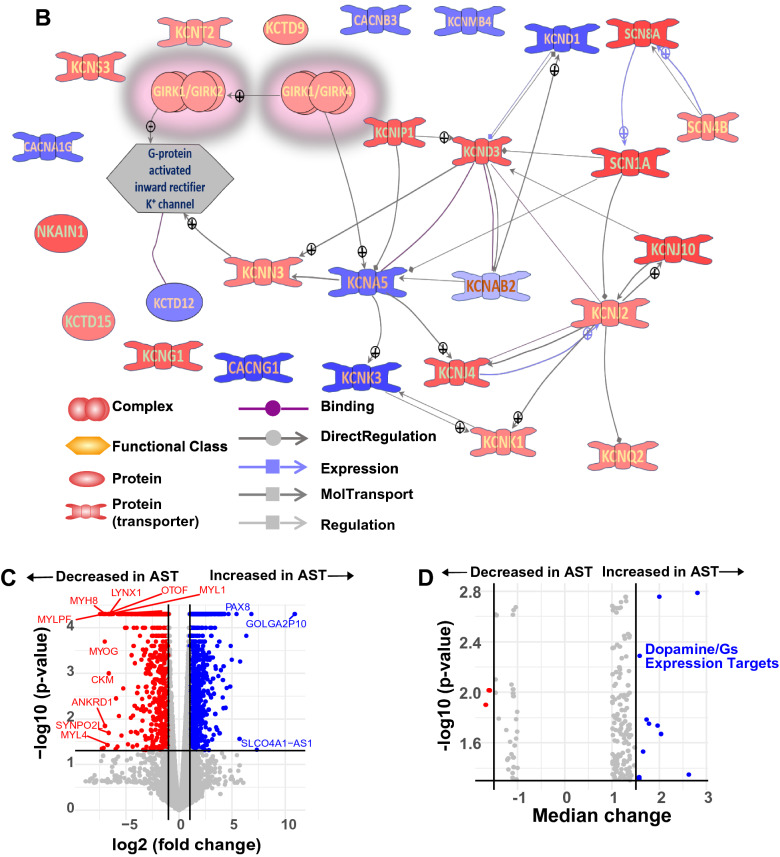

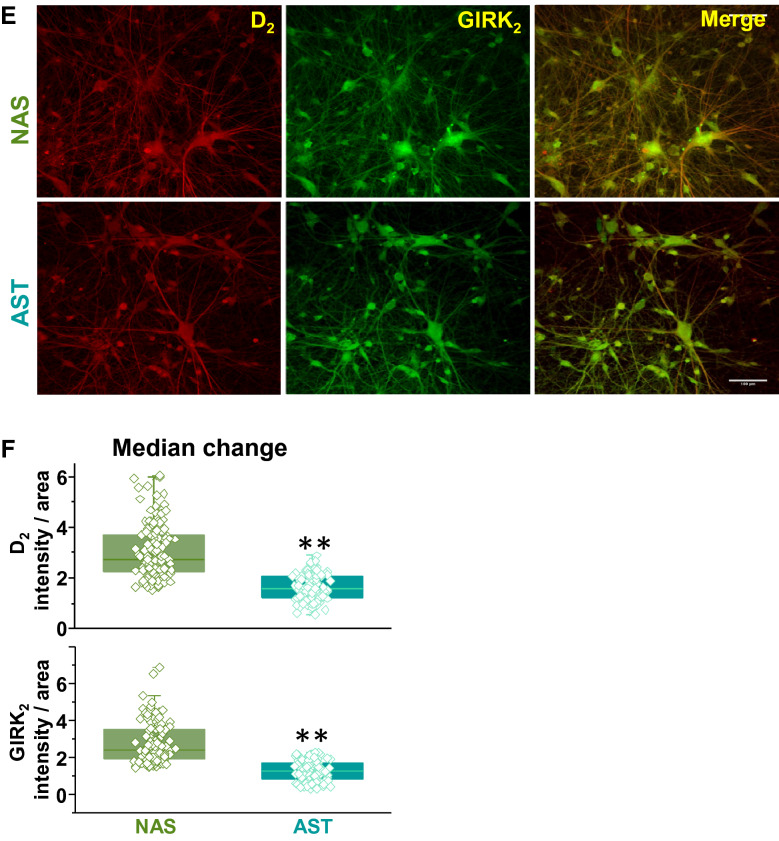
The transcriptome network and immunostaining for D2R and GIRK channels. **a** D2R pathway showed overall upregulation in AST-derived dopamine neurons with most elements in the D2R signaling cascade, such as Gβγ, increased in relative expression (red). Green indicates downregulation and gray indicates no change. Key to abbreviations provided in Additional file 3: Table S1. **b** A transcriptome network for GIRK in AST-derived neurons. Overall, there was an increase in this network. Red indicates that the transcript is increased in abundance and blue indicates that transcript is decreased in relative abundance compared to NAS. Gray indicates an entity or gene that was not measured or detected in derived neurons. Abbreviations are provided in B. **c** Analysis of bulk RNA sequencing of AST-derived dopamine neurons (n = 3) and NAS-derived dopamine neurons (n = 3) revealed 1512 differetially expressed genes with 916 decreased in AST-dervied dopamine neurons (log2(Fold change) < -1, *p* < 0.05) and 596 increased in AST-derived dopamine neurons (log2(Fold change > 1, *p* < 0.05). **d** Gene set enrichment analysis (GSEA) depicted as a volcano plot revealed genes associated with dopamine/Gs signaling were upregulated in AST-derived vs NAS-derived dopamine neurons (Median fold change > 1.5, *p* < 0.05). **e** Immunostaining for GIRK2 channel (K_ir_3.2) and D2R after 5 months differentiation of NAS and AST. **f** top. Parallel immunostaining via a blinded experimental design, revealed a lower K_ir_3.2 expression in AST-derived neurons than in NAS-derived neurons (t_257_ = 14.01, *p* < 0.001, two-tailed Student's t tests, n: NAS = 126, AST = 133). Bottom. Parallel immunostaining showed a reduced D2R expression in the AST-derived neurons compared with NAS-derived neurons (t_259_ = 13.59, *p* < 0.001, two-tailed Student's t tests, n: NAS = 136, AST = 125)

Because our electrophysiological data pinpointed the D2R-GIRK pathway being of particular interest, we employed RNA sequencing to identify putative transcripts involved in D2R signaling. Overall, we found many of the elements downstream of D2R were increased, including the Gβγ subunit, which regulates GIRK channel activity (Fig. 9a). Additionally, many of the affected pathways downstream from D2R converged on processes such as neurotransmitter uptake and synaptic transmission. We next examined the GIRK network and found that transcripts for many of the potassium channels related to GIRK complexes were upregulated in the AST-derived neurons, resulting in an overall decrease in GIRK1/2/4 complexes (Fig. 9b). The increase in the D2R pathway and increase in the GIRK network suggest a possible compensatory attempt due to loss of functional availability of the D2R autoinhibitory pathway.

Taking a more global approach, we found 1512 differentially expressed genes between the NAS and the AST-dervied dopamine neurons (Fig. 9c). 916 genes were decreased in the AST-derived dopamine neurons. Some of the most significantly regulated genes involved synaptic maintenance and cytoskeletal organization, consistent with the hypothesized role of α-synuclein in synaptic structure (Additional file 2: Data). Gene Set Enrichment Analysis (GSEA) revealed that one of the most regulated gene sets was the Dopamine/Gs Expression Targets (Fig. 9d), further supporting our functional data that dopamine receptor signaling is involved in AST-derived neuron dysfunction. Additional gene sets that were differentially regulated in the AST versus NAS groups included DRD1/5 expression targets and DRD3—> Dopamine Uptake (Additional file 3: Table S1). Overall, our transcriptomic analyses suggest that dopamine signaling is severely dysregulated in the AST-derived dopamine neurons and support the notion that the D2R-GIRK pathway may be disrupted.

We then sought to validate our findings at the protein level using double blinded and parallel immunostaining assays from three independent rounds of differentiation. We found that AST- and NAS-derived dopamine neurons express D2R and GIRK. Representative immunolabeling of D2R and GIRK in NAS- and AST-derived dopamine neurons are shown in Fig. 9e. There is lower a.u./μm^2^ immunolabeling for D2R in the AST-derived dopamine neurons (Fig. 9f, 1.6 ± 0.04 a.u./μm^2^, n = 125) compared to NAS-derived dopamine neurons (3.0 ± 0.1 a.u./μm^2^, n = 136, from three independent rounds of differentiation). Similarly, compared to NAS-derived dopamine neurons (2.8 ± 0.09 a.u./μm^2^, n = 126), AST-derived dopamine neurons show significantly lower a.u./μm^2^ immunolabeling for GIRK (1.3 ± 0.04 a.u. / μm^2^, n = 133, Fig. 9f). As described in literature derived from the Lovinger’s lab and other groups, the frequently used D2R and GIRK antibodies [27] are raised against the intracellular domain of these membrane proteins, therefore, the immunostaining data shown here are limited to identifying the total (cytoplasmic + membrane) D2R or GIRK levels. However, taken in the context of our earlier findings (Figs. 5, 6, 7, 8), these data support the overall notion that the D2R-GIRK pathway is functionally disrupted by increases in α-synuclein and that it represents a pharmacological target to restore normal neuronal activity.

### Single-neuron recordings show AST-derived dopamine neurons are less sensitive to D2R agonist and GIRK channel enhancer

**Fig. 10:**
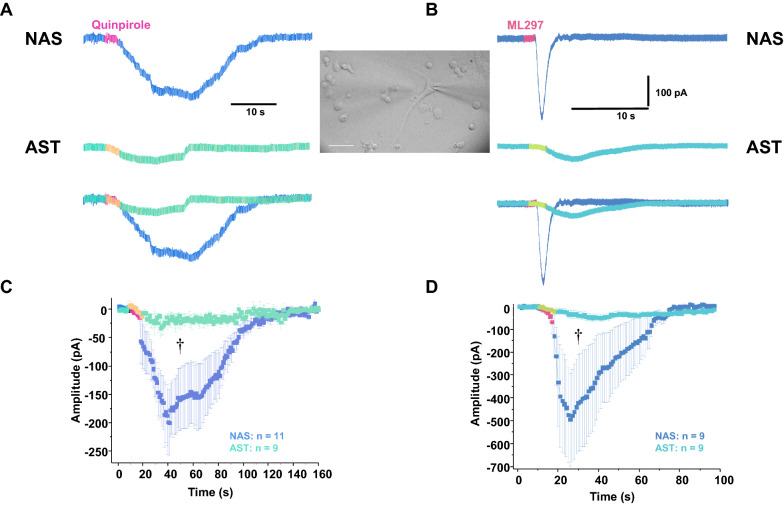
D2R- and GIRK-mediated currents in AST-derived dopamine neurons with α-synuclein triplication and NAS-derived dopamine neurons with normal α-synuclein levels. **a** Representative traces of single-neuron recordings after a brief (100 ms duration, 10 psi pressure, 50–100 μm application distance) focal application of quinpirole (5 μM) onto a NAS- (top panel) or an AST (middle panel)-derived dopamine neuron. The membrane voltage was held at − 60 mV in the voltage-clamp configuration. Superimposed representative traces (lower panel) show activation of D2R in an AST-derived dopamine neuron produced a distinctly smaller inward current, compared with the inward current measured in a NAS-derived dopamine neuron. **b** Representative traces of single-neuron recordings after a brief (100 ms duration, 10 psi pressure, 50–100 μm application distance) focal application of ML297 (5 μM) onto a NAS- (top) and an AST (middle)-derived dopamine neuron. Superimposed representative traces (bottom) show GIRK-mediated current in an AST-derived dopamine neuron was evidently smaller than that from a NAS-derived DA neuron. **c** The time course of quinpirole-induced inward currents was obtained by averaging 1 s interval activities at baseline and after quinpirole application. The inward currents in NAS-derived dopamine neurons were larger than in AST-derived dopamine neurons (†p < 0.05, Kolmogorov–Smirnov test). **d** The time course of ML297-induced GIRK-mediated currents was obtained by averaging 1 s interval activities at baseline and after ML297 application. The inward currents in NAS-derived dopamine neurons were larger than AST-derived dopamine neurons (†p < 0.05, Kolmogorov–Smirnov test). Inset A and B: A DIC image of a recording and a focal application pipettes during a patch-clamp experiment (scale bar, 50 μm). The analyses were performed via a blinded experimental design

In order to test our hypothesis that the D2R-GIRK pathway is functionally disrupted in AST-derived dopamine neurons, we performed complementary single-neuron recordings of D2R-mediated inward current. We measured D2R-mediated inward currents in the NAS- or AST- derived dopamine neurons after a brief (100 ms duration) focal application of quinpirole (5 μM, 100 ms duration, 10 psi pressure, 50–100 μm application distance). The membrane voltage was held at − 60 mV in the voltage-clamp configuration. The rapid application of quinpirole induced an inward current in NAS- or AST-derived neurons (Fig. 10a). The activation of D2R in the AST-derived dopamine neurons produced a significantly smaller inward current, compared with the inward current measured in the NAS-derived dopamine neurons (Fig. 10a, c, *p* < 0.05, Kolmogorov–Smirnov test). These data are consistent with our RNA sequencing data and the interpretation that the D2R-GIRK autoinhibitory pathway is functionally uncoupled, leading to decreased D2R-induced inward currents.

It has been shown that D2R activation increases GIRK conductance [12]. Therefore, we investigated whether the same stimulation protocol can generate GIRK-mediated current. We found a rapid and focal (100 ms duration, 10 psi pressure, 50–100 μm application distance) applications of 5 μM ML297, a GIRK activator, produced an inward current with spike-like shape in NAS-derived dopamine neurons (Fig. 10b). Similar to the D2R-medited currents, the size of ML297-induced GIRK-mediated current in AST-derived dopamine neurons was significantly smaller than those from NAS-derived neurons (Fig. 10b, d, *p* < 0.05, Kolmogorov–Smirnov test). Therefore, these data indicate that GIRK function is also decreased in AST-derived dopamine neurons, independent of D2R signaling. These findings collectively support the interpretation that α-synuclein overexpression decreases activities of both D2R signaling and GIRK channels.

### AST-derived dopamine neurons exhibited lower dopamine release

**Fig. 11:**
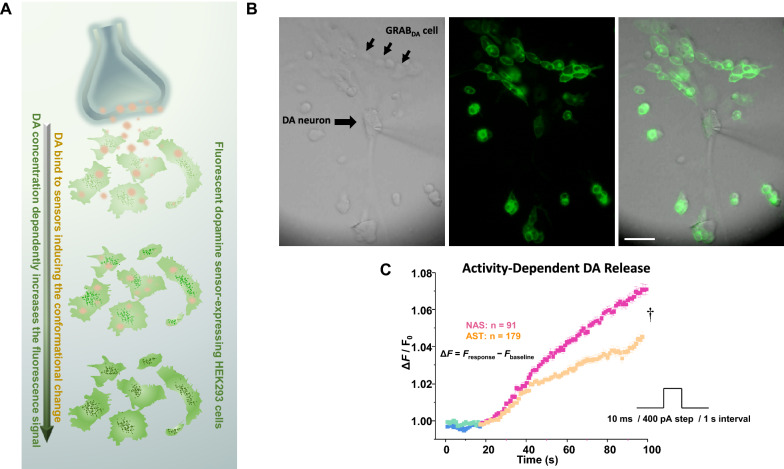
The depolarization-induced dopamine release is lower in AST-derived dopamine neurons. **a** Cartoon shows the experimental design for the detection of dopamine released from AST- or NAS-derived dopamine neurons. **b** The representative DIC image (left) shows: the stimulating electrode in cell-attached configuration, the neuron and sniffer cells (GRABDA4.4-expressing HEK293 cells) (middle) co-cultured with the neurons 24 h before experiments. The right panel shows the merged image (scale bar, 50 μm). **c** Via the cell-attached experimental configuration, a NAS- or AST-derived dopamine neuron was stimulated using a brief (10-ms) episodic (once every 1 s) depolarizing current (100 pA) pulse. The florescence intensity of sniffer cells rapidly increased following electrical stimulations, indirectly measuring fold change (Δ*F* /F_0_) in dopamine release from the neuron. The fluorescence signals were normalized to the averaged intensity of first 60 s for each experimental condition. The average florescence intensity of sniffer cells co-cultured with the AST-derived dopamine neuron was lower than those co-cultured with the NAS-derived dopamine neuron (†p < 0.05, Kolmogorov–Smirnov test). The experiments and analyses are performed via a blinded experimental design

The depolarization-induced dopamine release is one of the hallmarks of dopamine transmission [92]. The activity dependent properties of dopamine neurons allow them to optimize the release of dopamine in their terminal fields. Thus far, we have shown that both AST- and NAS-derived dopamine neurons have pacemaker activity, albeit with different patterns, that theoretically should lead to dopamine release. To measure depolarization-induced dopamine release we used GRAB_DA4.4_ (G protein-coupled receptor [GPCR]-activation-based) sensor-expressing HEK293 cells to measure extracellular dopamine levels. GRAB_DA4.4_ is a genetically encoded fluorescent dopamine sensor, engineered by coupling a conformationally-sensitive circular-permutated EGFP (cpEGFP) to D2 receptor. In GRAB_DA4.4_-expressing HEK293 cells (sniffer cells), dopamine binding to the sensor induces a conformational change which results in a robust increase in fluorescence signal in a concentration-dependent manner (Additional file 1: Fig. S5). We employed this approach to measure depolarization-induced changes in GRAB_DA4.4_ fluorescence signal when the GRAB_DA4.4_-expressing HEK293 cells were co-cultured with the NAS- or AST-derived dopamine neurons (Fig. 11). Notably, since the sensor is D2R-based, we co-cultured HEK293 cells expressing GRAB_DA4.4_ with the AST- and NAS-derived dopamine neurons to the possible confounder of differential D2R expression between the conditions. Via the cell-attached experimental configuration, a NAS- or AST-derived dopamine neuron was stimulated using a brief (10-ms) episodic (once every 1 s) depolarizing current (100 pA) pulse (Fig. 11b). The florescence intensity of sniffer cells (GRAB_DA4.4_-expressing HEK293 cells) rapidly increased following electrical stimulations, indirectly measuring dopamine release from the neuron (Fig. 11c). Employing an identical electrical stimulation protocol (10 ms, once every 1 s), the average florescence intensity of sniffer cells co-cultured with the AST-derived dopamine neuron was lower than those co-cultured with the NAS-derived dopamine neuron (Fig. 11c, *p* < 0.05, Kolmogorov–Smirnov test). Taken in the context of the decreases in TH, DAT, and Nrr1 transcripts in AST-derived dopamine neurons (Fig. 2b), our data collectively indicate that α-synuclein induces an increase in burst firing coupled to decrease in dopamine release per action potential.

### AST-derived dopamine neurons exhibit reduced arborization and larger soma area

**Fig. 12:**
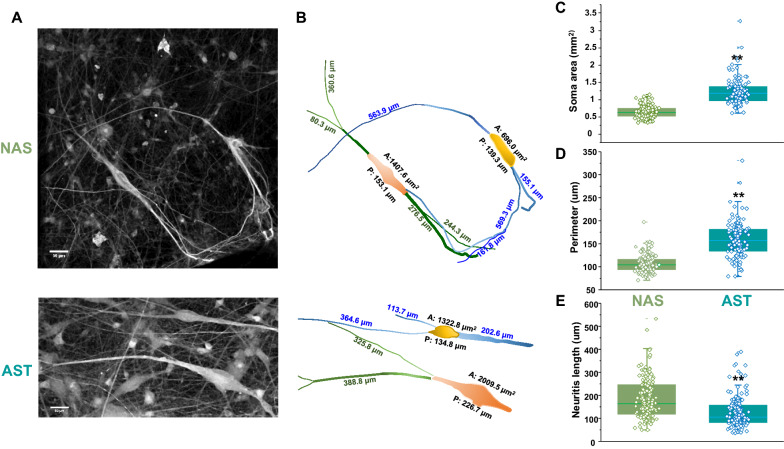
Morphological analysis after 5 months of differentiation revealed AST-derived neurons exhibit reduced arborization and larger soma area. The image analyses were performed via a blinded experimental design. **a** Representative images of NAS- and AST-derived neurons after 5 months of differentiation. **b** A schematic representation of the measurement from (**a**) by ImageJ. **c**, **d** Soma area and perimeter of AST-derived neurons are larger than NAS-derived neurons (Some area: t_331_ = -9.49, *p* < 0.001, two-tailed Student's t tests), (Perimeter: t_331_ = 14.89, *p* < 0.001, two-tailed Student's t tests). **e** Whereas, the neurite length of AST-derived neurons is shorter than NAS-derive neurons (t_310_ = 6.818, *p* < 0.001, two-tailed Student's t tests). n = 159 NAS/174 AST. Scale bar: 100 μm

α-synuclein has multiple characterized functions in neurons, such as sensing membrane curvature. It can also induce extensive membrane reshaping and membrane re-modelling [39, 95]. Morphological restructuring and decreased neuronal complexity have also been a hallmark of neuronal degeneration [17, 30, 81]. To examine whether increased α-synuclein affects overall neuronal arborization we measured soma area, neuronal perimeter, and neurite length in the AST- and NAS-derived human dopamine neurons. Morphological analyses of differentiated neurons revealed that AST-derived dopamine neurons exhibited larger soma area and perimeter than NAS-derived dopamine neurons (Fig. 12b, soma area, 712.3 ± 22.4 μm^2^ in NAS, vs. 1082.4 ± 29.7 μm^2^ in AST, *p* < 0.001, n = 159–174, from three independent rounds of differentiation. The perimeter comparison is shown in Fig. 12c, NAS: 111.3 ± 1.8 μm vs. AST: 161.4 ± 2.7 μm, p < 0.001, n = 159–174, from three independent rounds of differentiation). AST-derived dopamine neurons also had shorter neurites (Fig. 12d, NAS: 187.2 ± 7.5 μm vs. AST: 126.7 ± 3.7 μm, *p* < 0.001, n = 144, from three independent rounds of differentiation). The loss of neuronal complexity found in AST-derived dopamine neurons is consistent with post-mortem histopathological analysis in PD patients [65] suggesting decreased neuronal complexity and dendritic arborization precede neuronal death [17, 30, 81]. Thus, α-synuclein induces dysfunctional neuronal activity coupled to morphologic changes consistent with the events leading to degeneration.

## Discussion

In patients with α-synuclein triplication, the mechanisms culminating in progressive loss of dopamine neurons prior to clinical manifestation of Parkinson’s disease (PD) remain elusive. Human iPSCs-derived dopamine neurons from patients with inherited pathologies such as PD provide a clinically relevant model system to study pathophysiological manifestation of earlier development of PD prior to neuronal demise. The iPSC-derived dopamine neuron model system is applicable in biomedicine, specifically in the development of patient specific therapeutic strategies. In this study, we employed a previously used differentiation protocol to generate human-like dopamine neurons from a PD patient with α-synuclein triplication (AST) and an unaffected first-degree relative (NAS), serving as a control with a similar genetic background to minimize phenotypic differences. We found after four weeks of neuronal differentiation, both AST- and NAS-derived cells express TH, DAT and α-synuclein. However, these neuronal-like structures are silent; they do not exhibit spontaneous firing activity, a hallmark of dopaminergic neurons. We determined after a longer differentiation period of 150 days, both AST- and NAS-derived dopamine neurons not only expressed the canonical markers of dopaminergic neurons (Figs. 1, 2), but also exhibited spontaneous firing activity (Figs. 3, 4). Our functional analyses revealed striking observations of increased broadbrimmed spontaneous firing activity in the AST-derived dopamine neurons that is due to reduced D2R-mediated autoinhibition on these neurons. Our approach enables large-scale studies of an early pathophysiological manifestation of increased α-synuclein in dopamine neurons prior neuronal demise and provides a possible pharmacological target to alleviate neuronal dysfunction.

To investigate the nature of dopamine transmission before neuronal demise, we measured the activity of human-like dopamine neurons with or without increased α-synuclein levels, AST and NAS neurons respectively. Since the intrinsic firing behavior of cultured WT mouse dopamine neurons is well-established [46, 56, 75, 84], we compared the firing behavior of NAS-derived dopamine neurons to cultured WT mouse dopamine neurons with endogenous or overexpression of α-synuclein. Spike frequency for dopaminergic neurons is within a wide range that can be as low as 0.5 Hz or as high as 20 Hz [12, 46, 48, 52, 56, 66, 75, 76, 84, 108, 110, 124]. Consistent with the literature, we found both NAS-derived dopamine neurons and WT mouse dopamine neurons exhibited characteristic pacemaker-like firing activity and irregular burst firing pattern of 3–8 Hz. But, unexpectedly, the AST-derived human like dopamine neurons exhibited a unique pattern of spontaneous firing activity characterized by increased broadbrimmed firing bursts with a pause between subsequent broadbrimmed bursts and a noticeable up-state, all of which support the interpretation of reduced autoinhibitory modulation in these neurons.

In dopaminergic neurons, one of the autoinhibitory mechanisms is activation of D2 receptor-induced hyperpolarizing current via an activation of G-protein-coupled inwardly rectifying potassium (GIRK) channels. D2 receptors and GIRK channels can be coimmunoprecipitated [71], suggesting a functional interaction between the two proteins. Decreased membrane D2 levels, blockade of D2 receptors, desensitization of the receptor, or reduced receptor-effector coupling can lead to increased firing activity of dopamine neurons and the occurrence of the up-state. Neurons toggle between hyperpolarized (down-state) and depolarized (up-state) resting membrane potentials. In healthy dopamine neurons the up-state is only a few millivolts from the action potential threshold, and the membrane potential fluctuations around the up-state are of higher amplitude, whereas the down-state is relatively free of noise [73]. Reduced functional availability of D2 receptors can increase the amplitude of the up-state, augment the rate of firing frequency and enhance the size of bursts. The increased frequency and bursting can reciprocally activate D2 receptors, opening GIRK channels and increasing the membrane potassium conductance, thereby inhibiting dopamine neuron firing [12, 69, 102]. This results in long pause between subsequent broadbrimmed bursts and a noticeable up-state—the same pattern we identified in our AST-derived dopamine neurons. Our whole-cell patch clamp recordings combined with pharmacological manipulations such as application of a D2 receptor agonist (Figs. 5, 6) or a GIRK channel enhancer (Additional file 1: Fig. S3) confirmed the decreased functional availability of D2 receptors and GIRK channel activity in the AST-derived dopamine neurons. A D2 receptor agonist or the combinational application of D2 receptor agonist and a GIRK channel enhancer reinstated the firing activity to the levels measured in human-like dopamine neurons with endogenous levels of α-synuclein (Figs. 5, 6 and Additional file 1: Fig. S3).

If we presume a linear relationship between functional assays described above and the transcript levels in the AST-derived neurons, the results of our functional assays are consistent with altered GIRK channel transcript, but discordant with the qPCR data for D2 receptor (Fig. 2). In addition, while immunocytochemistry data show a lower signal for D2 receptor and GIRK (Fig. 9f), we are hesitant to make any assumption about lower membrane levels of these proteins. This is because the commonly used and validated antibodies for D2 and GIRK are raised against the intracellular domain of these proteins that label both membrane and intracellular D2 receptors or GIRK channels [26, 27]. Similar technical limitations apply to the single cell qPCR assay, where total transcript levels do not necessarily reflect functional D2 receptors or GIRK channels at the membrane, receptors desensitization or altered receptor-effector coupling [9, 68, 107]. The complex interactions among D2 receptor signaling, GIRK channels and other relevant ion channels becomes more apparent with RNA-seq analysis. Therefore, live cell functional assays, such as electrophysiology, combined with pharmacological manipulations may be more reliable strategies to identify early pathophysiological manifestation of neuronal dysfunction in the AST-derived dopamine neurons. Nevertheless, a comprehensive, integrative investigation at the molecular, physiological, and morphological level will yield the most complete view of dopamine neuron dysfunction.

The notion that dopamine receptors’ expression or function are altered in PD has been shown frequently in both humans and in animal models of PD. Drug-naïve PD patients have been reported to have increased D2-like [72] and decreased D3-like [16] post synaptic dopamine receptors. Notably these studies were conducted in post-mortem tissue or in clinically diagnosed PD patients. Our data indicate that pre-synaptic D2R dysfunction may precede neuronal demise and the subsequent alterations in post-synaptic dopamine receptors, providing an added layer of complexity to studies on prodromal PD. Corroborating the notion that pre-synaptic dopamine receptors may be affected in PD, studies in LRRK2 mutant iPSC lines and animal models have shown D2R and D3R exhibit altered expression, localization, or function compared to control condition [18, 93, 104, 126]. The consistent implication of dopamine receptor dysfunction across different forms of autosomal dominant PD suggests that alteration in dopamine receptors may represent a common pathophysiological pathway preceding dopamine neurodegeneration.

The literature supports the interpretation that increased α-synuclein in dopamine neurons lead to dysregulation of dopamine transmission. In mouse overexpressing human α‐synuclein, Lam and colleagues reported an elevated tonic extracellular dopamine concentration that precedes loss of dopaminergic neurons followed by the eventual decline in dopamine levels due to neuronal degeneration [70]. Consistent with this report, we and others have shown α-synuclein overexpression increases extracellular dopamine levels by decreasing dopamine uptake and a five-fold increase in dopamine transporter mediated dopamine efflux [22, 116, 130, 134]. Decreased dopamine recycling leads to increased extracellular dopamine levels [45], downregulation of D2 auto-receptor activity, and increased firing activity of the neuron, but a lower amount of dopamine release per action potential, due to decreased tissue dopamine levels. Our simultaneous electrophysiology and live-cell imaging data using GRAB-_DA4.4_ revealed attenuation of dopamine release upon membrane depolarization. Therefore, it is possible that at early, premanifest stage of PD, there is a hypo-dopaminergic state inside the neuron, but a transient hyper-dopamine state extracellularly.

We showed that human and mouse dopaminergic neurons with normal levels of α-synuclein fire in tonic pacemaker fashion in striking contrast to the observed burst firing modes in the presence of increased α-synuclein (both AST-derived human-like dopamine neurons and mouse dopamine neurons overexpressing α-synuclein). Burst firing is observed periodically in healthy dopaminergic neurons and it requires significant bioenergetic resources [10, 46, 112, 135]. To sustain persistent burst firing patterns, dopaminergic neurons must replenish vesicular storage [41], recycle and synthesize protein [14, 57], reuptake neurotransmitter, (via a secondary activate and voltage dependent mechanism) [22, 123] and repolarize their membrane [33]. These innate mechanisms require increased oxygen [62], glucose [118], and ATP consumption and thereby present a metabolic burden on mitochondria [60, 77, 79]. However, in a healthy state, the burst firing occurs transiently, therefore, neuronal homeostasis is maintained. The persistent bursts in α-synuclein overexpressing neurons may lead to increased metabolic costs that can cascade to structural and functional detriments within the neuron, such as loss of neuronal complexity [86]. This interpretation is supported by recent reports showing the extensive arborization of dopaminergic neurons is linked to neuronal vulnerability [44], which is a key contributor of metabolic costs [95]. Therefore, we propose that increases in α-synuclein induce persistent bursting that imposes bioenergetic constraints within the neuron, and subsequently results in size alterations, loss of key proteins, and eventual loss of dopaminergic tone as the neuron attempts to compensate. Intervening with a D2R agonist to rescue the altered firing activity may then represent a promising strategy to forestall neuronal demise.

## Materials and methods

### iPSCs and maintenance culture

Via a material transfer agreement between University of Florida and University of Edinberg, we received the iPSCs of a member of the Iowa kindred carrying a triplication of the SNCA locus and an unaffected member of the family without this mutation from Dr. Tilo Kunath laboratory [32].

Mitotically inactivated mouse embryonic feeders (MEFs, ThermoFisher Scientific) were seeded onto gelatin‐coated tissue culture plates at a density of 2.6 × 104 cells per cm^2^ in DMEM-F12 (ThermoFisher Scientific), with 10% fetal bovine serum, 1 mM GlutaMax, 0.1 mM nonessential amino acids, 100 U/ml penicillin, 100 μg/ml streptomycin. Two days after seeding, the medium was replaced with 80% knockout DMEM, 20% knockout serum replacer, 1 mM GlutaMax, 0.1 mM nonessential amino acids, 100 U/ml penicillin, 100 μg/ml streptomycin (all above from Thermo Fisher Scientific Life Sciences, Waltham, MA), and 4 ng/ml fibroblast growth factor (FGF-basic, Peprotech, Cranbury, NJ). iPSCs (passages 30–36), were seeded onto the MEFs, and colonies were manually passaged once every 8–10 days using a fire‐polished glass Pasteur pipette. Media were changed every 48 h and cells were maintained at 37 °C/5% CO^2^.

### Dopamine neuron differentiation from iPSCs

Cells were plated (35 × 103–40 × 103 cells per cm^2^) and grown for 5 days on matrigel (Corming)-coated dishes in knockout serum replacement medium (KSR, Life Technologies, Grand Island, NY) containing Dulbecco's Modified Eagle Medium/Nutrient Mixture F-12 (DMEM/F12, Life Technologies, Grand Island, NY), 15% knockout serum replacement, 50 nM PD173074 (Stemcell, Vancouver, Canada), 200 ng/ml Noggin (Stemcell, Vancouver, Canada), 100 nM LDN193189 (Stemcell, Vancouver, Canada), 10 µM SB431542 (Tocris, Minneapolis, MN), 500 ng/ml Shh (R & D), 2 µM Purmorphamine (ReproCell, Beltsville, MD), 0.7 µM CHIR99021(Peprotech, Cranbury, NJ), 10 µM Y-27632 (Peprotech, Cranbury, NJ). From day 5 to day 11, KSR containing medium was replaced with N2 (Life Technologies, Grand Island, NY) medium. PD173074 and Shh were withdrawn at day 8,but added 1% N2 and 100 ng/ml FGF8a (R & D). On day 12, media was changed to balanced neuronal medium/B27 containing medium (B27; Life Technologies, Grand Island, NY) supplemented with CHIR (until day 13) and with 0.5 mM DcAMP, 200 ng/ml Noggin, 100 nM LDN193189, 10 µM SB431542 for 10 days. Cells were dissociated using Accutase (Innovative Cell Technology, San Diego, CA) and replanted under high cell density conditions (300 × 103–400 × 103 cells per cm^2^) on 12 mm round coverslips coated with 100 μg/ml poly-l-lysine and 5 μg/ml laminin in 35 × 10 mm tissue culture Petri dishes in N2 medium supplemented with B27, 200 μM L-Ascorbic acid, 20 ng/ml BDNF, 20 ng/ml GDNF, 0.5 mM dibutyryl cAMP (all from Millipore Sigma), 1 ng/ml TGF-β3 (R & D). (Fig. 1).

### Quantitative real-time PCR

Total RNA was extracted using a RNeasy kit (Qiagen). For each sample, 1 μg of total RNA was treated for DNA contamination and reverse transcribed using the Quantitect RT kit (Qiagen). Amplified material was detected using Quantitect SYBR green probes and PCR kit (Qiagen) on a Mastercycler RealPlex2 (Eppendorf). All results were normalized to a HPRT control and are from 4 to 6 technical replicates of 2–3 independent biological samples at each data point.

### RNA-seq and pathway analysis in dopamine neurons differentiated from iPSCs

RNA concentration was determined using the Qubit® 2.0 Fluorometer (ThermoFisher/Invitrogen, Grand Island, NY), and RNA quality was assessed using the Agilent 2100 Bioanalyzer (Agilent Technologies, Inc.). RNA samples with a measured 28S/18S > 1 and RNA integrity number (RIN) ≥ 7 were used for RNAseq library construction. RNA-seq library were constructed using the NEBNext® Ultra™ Directional RNA Library Prep Kit for Illumina (New England Biolabs, USA) following manufacturer’s recommendations. Approximately 125 ng of total RNA was used for mRNA isolation using the NEBNext Ploy(A) mRNA Magnetic Isolation module (New England Biolabs, USA). RNA library construction was then performed with the NEBNext® Ultra™ II Directional RNA Library Prep Kit for Illumina® (New England Biolabs, catalog #E7760) according to the manufacturer's user guide. Barcoded libraries were sized on the bioanalyzer and quantitated by QUBIT. Six individually prepared libraries (n = 3 for AST and n = 3 for NST) were pooled by equimolar and sequenced by Illumina HiSeq 3000 2X100 cycles run (Illumina Inc., CA, U.S.A). RNA library construction and sequencing were performed at the Interdisciplinary Center for Biotechnology Research (ICBR) Gene Expression and Genotyping Core, University of Florida (UF).

Reference based transcriptome analysis was conducted for the six samples. The reference genome used was GRCh38.p10 Genome Reference Consortium Human Build 38 patch release 10 (GRCh38.p10), downloaded from the Ensemble database. From a total of 152.85 million reads, 149.45 million high quality reads were used in downstream analysis. An average of 91.47% of the reads aligned to the reference genome. An average of 83,119 transcripts and 22,485 genes were expressed across all samples. All quality control data are provided in Additional file 2: Data.

The raw data generated was first assessed for quality using FastQC (Babraham Bioinformatics). Reads were pre-processed to remove the adapter sequences and removal of the low-quality bases (< q30). Pre-processing of the data was done with Cutadapt [85]. An average of 97% of high-quality reads were retained for the downstream analysis. HISAT2 [63], a splice aligner program, was used to align the sequencing data to the human reference genome using the default parameters. HISAT2 is a fast and sensitive alignment program for mapping next-generation sequencing reads (both DNA and RNA) to a population of human genomes (as well as to a single reference genome). Reads were then classified into aligned reads (which align to the reference genome) and unaligned reads. Cufflinks [127] was used to estimate and calculate transcript abundance and normalized read count using Fragments Per Kilobase of transcript per Million mapped reads (or FPKM). Cuffdiff [127] was then used to identify the differentially expressed transcripts expressed as log2fold change values. Sequencing files and expression data have been deposited in Geo NCBI (accession number: GSE163344). All expression data are provided in Additional file 2: Data.

Pathway analysis was conducted in Pathway Studio 12.3 (Elsevier). Human genes were mapped to the mammalian database using the official gene Name + Alias. Gene set enrichment analysis proceeded with 1000 permutations to generate the distributions for statistical testing (permutation test). Gene sets identified as enriched in AST compared to NST were those with a *P* < 0.05. All GSEA data are provided in Additional file 2: Data along with abbreviations for gene networks presented. Normalized gene expression values, and gene set enrichment data were then plotted as volcano plots using R statistical software.

### Preparation of primary mouse midbrain dopaminergic neuronal culture

Mice were housed in the animal care facilities at the University of Florida in accordance with Institutional Animal Care and Use Committee, under guidelines established by National Institutes of Health. Food and water were available ad libitum in the home cage. Animals were housed under standard conditions at 22 − 24 °C, 50–60% humidity, and a 12 h light/dark cycle. Primary mouse midbrain dopamine neuron culture was prepared as described previously [75, 76]. Midbrain regions from P0 mice were dissected, dissociated, and plated on Poly-l-lysince- and laminin-treated coverslips. Cells were maintained at 37 °C in a 5% CO_2_ humidified incubator with a culture medium consisting of Neurobasal (Life Technologies, Grand Island, NY), 0.9% L-glutamine, 2% B27 and 1 ng/ml GDNF.

### Electrophysiological recordings

Whole-cell recordings: Spontaneous firing activity of midbrain dopamine neurons was examined via whole cell current clamp recordings. The neurons were continuously perfused with aCSF containing the following (in mM): 126 NaCl, 2.5 KCl, 2 CaCl_2_, 26 NaHCO_3_, 1.25 NaH2PO_4_, 2 MgSO_4_, and 10 dextrose, equilibrated with 95% O_2_/5% CO_2_; pH was adjusted to 7.4 at 37 °C. Patch electrodes were fabricated from borosilicate glass (1.5 mm outer diameter; World Precision Instruments, Sarasota, FL) with the P-2000 puller (Sutter Instruments, Novato, CA). The tip resistance was in the range of 3–5 MΩ. The electrodes were filled with a pipette solution containing (in mM): 120 potassium-gluconate, 20 KCl, 2 MgCl_2_, 10 HEPES, 0.1 EGTA, 2 ATP, and 0.25 GTP, with pH adjusted to 7.25 with KOH. All experiments were performed at 37 °C. To standardize action potential (AP) recordings, neurons were held at their resting membrane potential (see below) by DC application through the recording electrode. AP was recorded if the following criteria were met: a resting membrane potential of less than − 35 mV and an AP peak amplitude of > 60 mV. AP half-width was measured as the spike width at the half-maximal voltage using Clampfit 10 software (Axon instruments, Foster City, CA). Steady-state basal activity was recorded for 2–3 min before bath application of the drug. For experiments involving drug application. The spontaneous spike activity of midbrain dopamine neurons was obtained by averaging 1 min interval activities at baseline (before drug) and after 7–10 min of drug.

The series resistances were in the range of 5–10 MΩ (typically 5 MΩ) and were compensated 60% on-line. Membrane potential measurements were not corrected for the liquid junction potential (∼15 mV). Leak currents were subtracted using a standard P/4 protocol. Before seals (5 GΩ) were made on cells, offset potentials were nulled. Capacitance subtraction was used in all recordings.

### DopamineD_2_ receptor- and GIRK-mediated inward currents recording

The transient focal application of agonists was exploited to evoke currents. A pneumatic pico-pump (PV830, WPI, Sarasota, FL) was used for quinpirole or ML 297 delivery via a pipette (3–5 MΩ) identical to that used for patch-clamp recordings. For currents generation, a glass pipette was placed within 50–100 μm of the soma, 10 psi pressure and 50–100 ms duration were applied to activate the D2R or GIRK channel. Whole-cell recordings for inward currents were obtained at − 60 mV holding potential using an internal solution described above.

### Immunofluorescence staining and confocal imaging

The neurons were grown on glass coverslips as described above. The neurons were then washed with HBSS solution (Life Technologies, Grand Island, NY) and fixed with freshly prepared 3.7% paraformaldehyde (Electron Microscopy Sciences, Hatfield, PA) for 20 min at room temperature and washed twice with PBS solution. For immunolabeling, the cells were then permeabilized as described previously [22, 75, 94, 110]. Washes, blocking, and incubation with primary and secondary antibodies were carried out in cell collection chambers on the stage of an orbital shaker. The neurons were incubated in blocking solution containing 10% normal goat serum (LAMPIRE Biological Laboratories, Pipersville, PA) and 0.5% Triton X-100 in PBS (Sigma,St. Louis, MO) at room temperature for one hour. The neurons were then incubated with a solution containing primary antibody (1:1000), 0.1% Triton X-100 and 5% normal goat serum at 4 °C overnight. On the following day, the primary antibody was removed, and cells were subjected to three 20 min washes prior to addition of Alexa-Fluor 488- or 647 conjugated secondary antibodies (Life Technologies, Grand Island, NY) diluted at 1:200–500 in PBS. Cells were incubated in secondary antibody for one hour in the dark, at room temperature followed by three 20 min washes. Coverslips were then mounted on the Superfrost Excell Microscope Slides (VWR,West Chester, PA) using Flouromount-G (SouthernBiotech, Birmingham, AL). Slides were stored in the dark at 4 °C until imaging. Confocal images were collected using a Nikon A1 laser-scanning confocal microscope. Excitation wavelengths were set at 488 and 647-nm, for the respective fluorescent markers. Images were acquired using 488-nm excitation with a 514-nm long pass filter, 647- nm excitation with a 668-nm long pass filter. Images were taken using a 20X or a 60X Nikon objective with 1.40 NA (numerical aperture).

All imaging analyses were carried out via an automated graphic plugin for the public domain image analysis software ImageJ (Wayne Rasband; Research Services Branch, National Institutes of Mental Health, National Institutes of Health, Bethesda MD). The morphological analysis were performed from DIC images. For RGB images, average gray value within the selection as image intensity is the sum of the gray values of all the pixels in the selection divided by the number of pixels. The mean is calculated by converting each pixel to grayscale using the formula gray = (red + green + blue)/3. Area of selection was in square pixels or in calibrated square units (μm^2^). The perimeter of a composite selection is calculated by decomposing it into individual selections. The Neurite Tracing is based on multiscale image processing using both first-order (edge) and second-order (ridge) filters and combining the extracted information in a cost function that is globally minimized algorithms to detect elongated image structures and determine their centerlines [88]. The images were processed using ImageJ and the NeuronJ plugin for ImageJ which highlights and traces neurites and somas [49, 101]. The trace was manually initiated and ended of each neurite, using a crosshair pointer, and map the length and shape of each neurite with a series of connected straight lines. The tracing algorithm computed the optimal path between the selected starts and end points. Soma size was measured using the “selection brush” tool on ImageJ to carefully outline the shape of the soma. Requirements for counting a projection as a dendrite included: (1) Neurons being analyzed were at least one soma away from another neuron. (2) Projections were counted as dendrites if the length were at least the diameter of the soma. Fluorescence colocalization analysis was used to determine whether two proteins associate with the same subnuclear structures or with the same plasma membrane domains. We used three of frequently used colocalization coefficients to express the intensity correlation of colocalizing objects in each component of a dual-color image (see Additional file 2: Data): (1) Pearson's correlation coefficient: The Pearson's correlation coefficient is not sensitive to differences in mean signal intensities or range, or a zero offset between the two components. The result is + 1 for perfect correlation, 0 for no correlation, and − 1 for perfect anti-correlation. Noise makes the value closer to 0 than it should be [97]. (2) Spearman's rank correlation coefficient: The Spearman correlation between two variables is equal to the Pearson correlation between the rank values of those two variables, while Pearson's correlation assesses linear relationships, Spearman's correlation assesses monotonic relationships (whether linear or not). If there are no repeated data values, a perfect Spearman correlation of + 1 or − 1 occurs when each of the variables is a perfect monotone function of the other [120]. (3) Kendall rank correlation coefficient: the Kendall correlation between two variables will be high when observations have a similar (or identical for a correlation of 1) rank (i.e., relative position label of the observations within the variable: 1st, 2nd, 3rd, etc.) between the two variables, and low when observations have a dissimilar (or fully different for a correlation of − 1) rank between the two variables. Contrary to the Spearman correlation, the Kendall correlation is not affected by how far from each other ranks are but only by whether the ranks between observations are equal or not and is thus only appropriate for discrete variables but not defined for continuous variables [61].

### Measurement of dopamine release

Dopamine release was measured by via changes in the GRAB_DA4.4_ fluorescence signal in HEK293 cells stably expressing GRAB_DA4.4_. The GRAB_DA4.4_-expressing cells were generously gifted by Dr. Ulrik Gether. The dopamine sensor GRAB_DA4.4_ is a genetically encoded fluorescent dopamine sensor, engineered by coupling a conformationally-sensitive circular-permutated EGFP (cpEGFP) to D2R. In GRAB_DA4.4_-expressing HEK293 cells, dopamine binding to the sensor induces a conformational change which results in a robust increase in fluorescence signal via a concentration-dependent manner [122]. The GRAB_DA4.4_-expressing HEK 293 cells were maintained in DMEM supplemented with 10% FBS and 1% Penicillin/Streptomycin. Selection pressure for GRAB_DA4.4_ expressing cells was maintained with media containing Hygro-B (1 mg/ml), Blasticidin (0.015 mg/ml), and tetracycline (1:1000). 24 h before measurement of dopamine release, equal number of GRAB_DA4.4_ expressing cells were plated on coverslips harboring either NAS- or AST-derived dopamine neurons**.**

Induction of dopamine release was performed via episodic current stimulations (once every 1 s) in cell-attached or after micro-focal application of agonists when the neuron was held in the whole-cell configuration. To electrically trigger dopamine release, the stimulation pipette was pulled from thin-walled borosilicate glass capillaries (1.5-mm outer diameter; World Precision Instruments, Sarasota, FL) to a final resistance of 3–4 MΩ and filled with aCSF. A MultiClamp 700B amplifier (Molecular Devices, Sunnyvale, CA, USA) was used to obtain cell-attached configuration. Seal resistance in the cell-attached mode was > 4 GΩ. An episodic (once every 1 s) and 10-ms depolarizing current pulse (100 pA) from the holding potential of − 60 mV was applied. To pharmacologically induce dopamine release current stimulations was not applied.

Blue light (470 nm) laser illumination was applied for 70 ms using a Digital Mirror Device based pattern illuminator (Mightex Polygon 400, Mightex Systems). For quantification of fluorescence intensity of GRAB_DA4.4_-expressing HEK293 cells, experimenter-defined ROIs were created for each cell to exclude both the overlap between adjacent cells and measurement of intensity around the membrane of the cells near the neurons. Background fluorescence was subtracted from all images. Mean intensity over time for each ROI was recorded continuously before and after electronic or focal application (via Pico-spitzer) of agonists. Experiments were performed in the isotonic, isosmotic external solution described above. The baseline fluorescent intensity is defined as the average fluorescent intensity 1 min (Fig. 11 and Additional file 1: Fig. S5). All values were normalized to the baseline fluorescent intensity. Unless indicated, all electrophysiological and live-imaging experiments were performed in the presence of the following antagonists in the aCSF: 10 μM SR-95531 (GABAA receptor), 100 nM CGP 35,348 (GABAB-receptor), 20 μM MK-801(NMDA glutamate receptor), 10 μM CNQX (AMPA/kainate glutamate receptor) and 1 μM SCH-23390 (dopamine D_1_ receptor).

### Statistical and data analysis

The electrophysiology data was acquired using the Clampex 10 software package (Axon Instruments, Foster City, CA). The data were analyzed offline using pClamp 10. For all experiments, the data are presented as mean ± SEM. N denotes the number of neurons or cells for each experiment. Statistical significance was assessed using two-tailed Student's t tests or one-way ANOVA. If ANOVA showed statistical significance, all pairwise post hoc analysis was performed using a Tukey's post hoc test. Differences were considered significant at *P* < 0.05. * denotes significance < 0.05. ** denotes significance < 0.01. The coefficient of variation is a measure of the relative spread of the data. It is computed as the standard deviation divided by the mean times 100%.

## Supplementary Information

**Additional file 1:** Supplemental figure 1–5.

**Additional file 2:**  Results for supplemental data.

**Additional file 3:** Gene sets enriched for dopamine signaling and processing.
