# Supplementary material for: In Parkinson's patient-derived dopamine neurons, the triplication of α-synuclein locus induces distinctive firing pattern by impeding D2 receptor autoinhibition
Source: Acta Neuropathol Commun. 2021 Jun 7;9:107. doi: 10.1186/s40478-021-01203-9 (PMC8185945; doi:10.1186/s40478-021-01203-9)
Supplement: Supplementary file 1 — Additional file 1: Supplemental figure 1–5. [file 40478_2021_1203_MOESM1_ESM.pptx]

## Slide 1
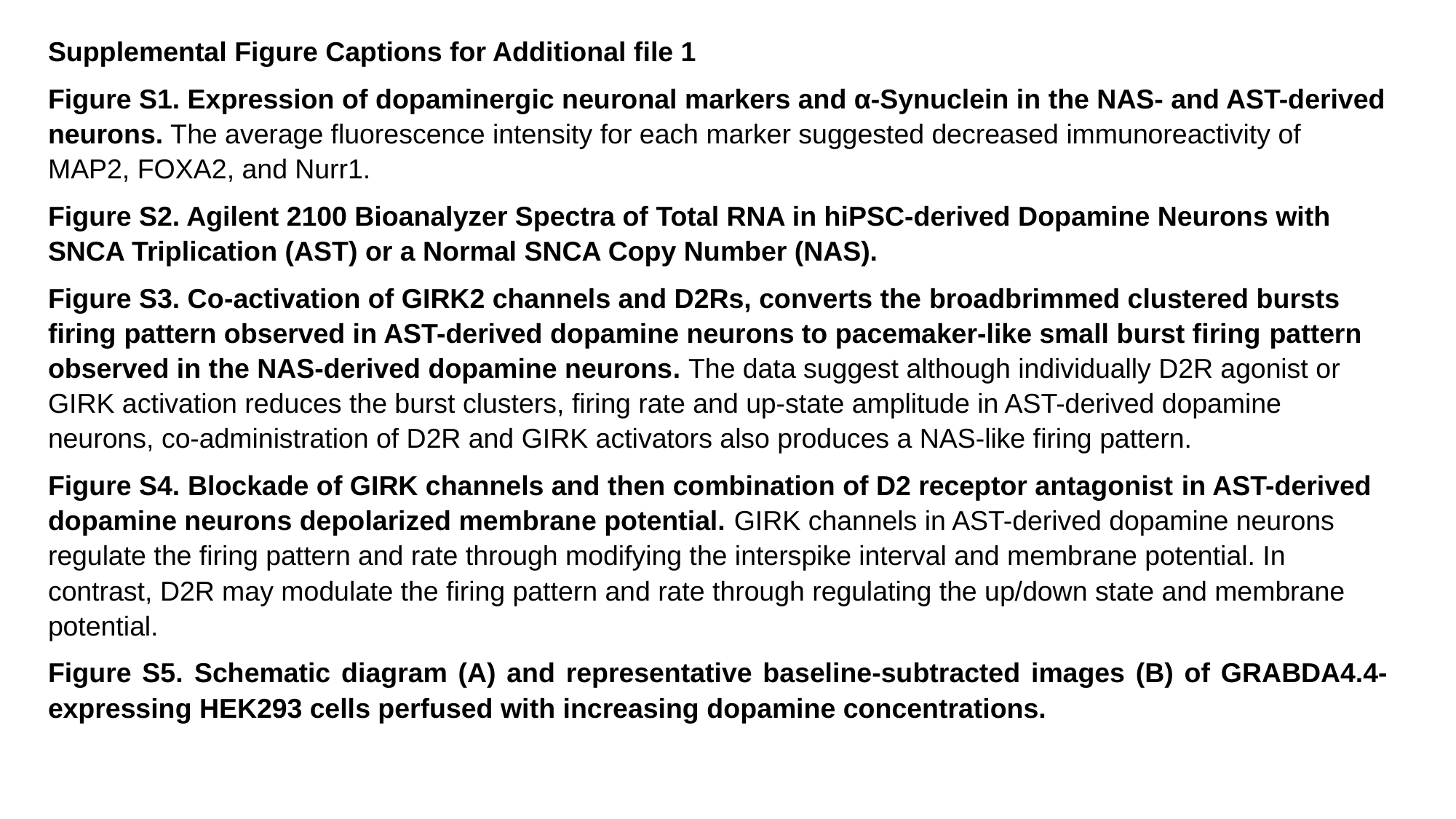

Supplemental Figure Captions for Additional file 1
Figure S1. Expression of dopaminergic neuronal markers and α-Synuclein in the NAS- and AST-derived neurons. The average fluorescence intensity for each marker suggested decreased immunoreactivity of MAP2, FOXA2, and Nurr1.
Figure S2. Agilent 2100 Bioanalyzer Spectra of Total RNA in hiPSC-derived Dopamine Neurons with SNCA Triplication (AST) or a Normal SNCA Copy Number (NAS).
Figure S3. Co-activation of GIRK2 channels and D2Rs, converts the broadbrimmed clustered bursts firing pattern observed in AST-derived dopamine neurons to pacemaker-like small burst firing pattern observed in the NAS-derived dopamine neurons. The data suggest although individually D2R agonist or GIRK activation reduces the burst clusters, firing rate and up-state amplitude in AST-derived dopamine neurons, co-administration of D2R and GIRK activators also produces a NAS-like firing pattern.
Figure S4. Blockade of GIRK channels and then combination of D2 receptor antagonist in AST-derived dopamine neurons depolarized membrane potential. GIRK channels in AST-derived dopamine neurons regulate the firing pattern and rate through modifying the interspike interval and membrane potential. In contrast, D2R may modulate the firing pattern and rate through regulating the up/down state and membrane potential.
Figure S5. Schematic diagram (A) and representative baseline-subtracted images (B) of GRABDA4.4-expressing HEK293 cells perfused with increasing dopamine concentrations.

## Slide 2
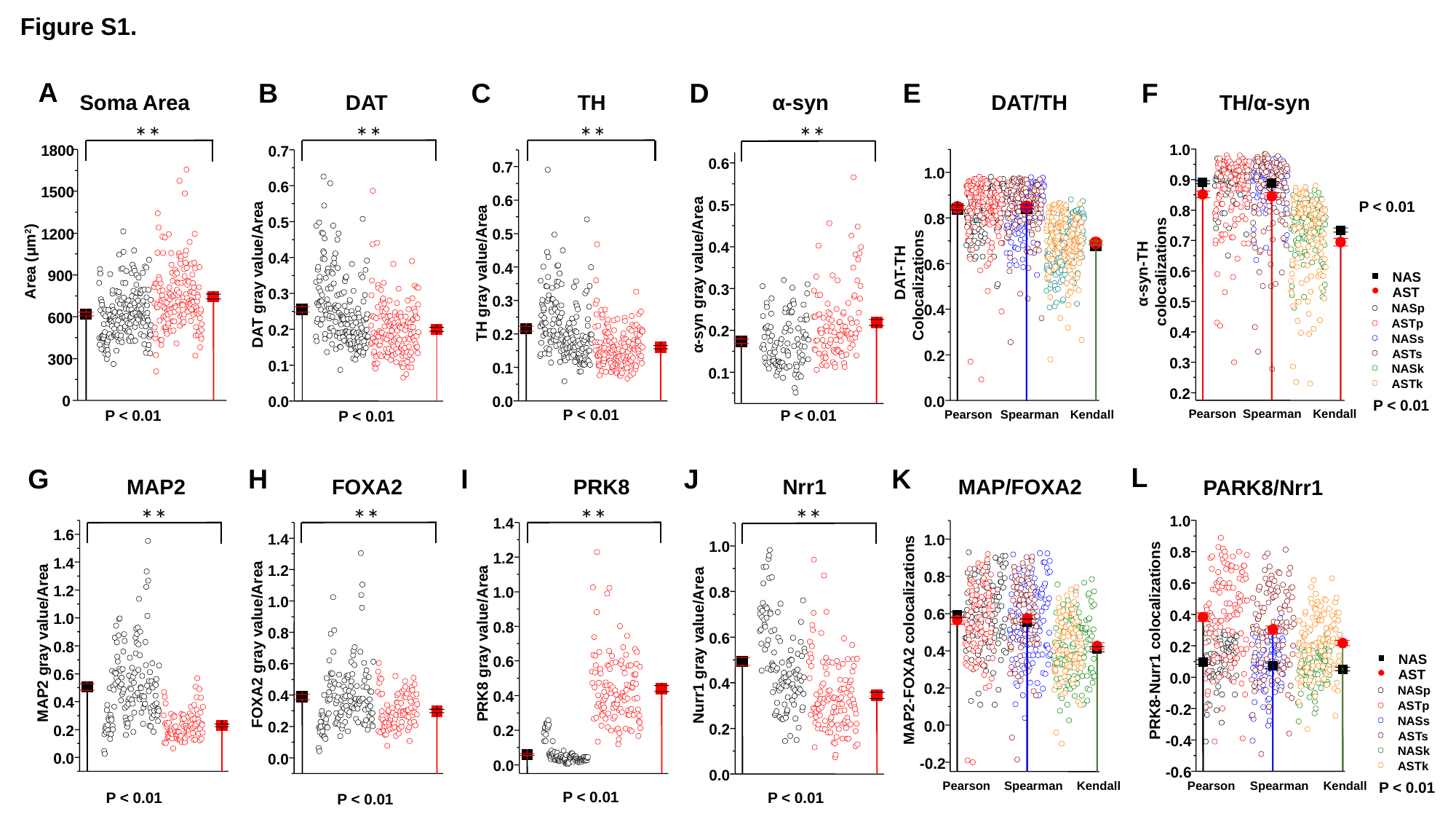

Figure S1.
A
B
C
D
E
F
Soma Area
DAT
TH
α-syn
DAT/TH
TH/α-syn
**
**
**
**
1.0
0.9
0.8
0.7
α-syn-TH
 colocalizations
0.6
0.5
0.4
0.3
0.2
Pearson
Spearman
Kendall
1800
1500
1200
Area (μm2)
900
600
300
0
0.7
0.6
0.5
0.4
DAT gray value/Area
0.3
0.2
0.1
0.0
1.0
0.8
0.6
 DAT-TH
Colocalizations
0.4
0.2
0.0
Pearson
Spearman
Kendall
0.7
0.6
0.5
0.4
TH gray value/Area
0.3
0.2
0.1
0.0
0.6
0.5
0.4
α-syn gray value/Area
0.3
0.2
0.1
P < 0.01
NAS
AST
NASp
ASTp
NASs
ASTs
NASk
ASTk
P < 0.01
P < 0.01
P < 0.01
P < 0.01
P < 0.01
L
G
H
I
J
K
MAP/FOXA2
MAP2
FOXA2
PRK8
Nrr1
PARK8/Nrr1
**
**
**
**
1.0
0.8
0.6
0.4
PRK8-Nurr1 colocalizations
0.2
0.0
-0.2
-0.4
-0.6
Pearson
Spearman
Kendall
1.4
1.2
1.0
0.8
PRK8 gray value/Area
0.6
0.4
0.2
0.0
1.6
1.4
1.2
1.0
MAP2 gray value/Area
0.8
0.6
0.4
0.2
0.0
1.0
0.8
0.6
MAP2-FOXA2 colocalizations
0.4
0.2
0.0
-0.2
Pearson
Spearman
Kendall
1.4
1.2
1.0
0.8
FOXA2 gray value/Area
0.6
0.4
0.2
0.0
1.0
0.8
0.6
Nurr1 gray value/Area
0.4
0.2
0.0
NAS
AST
NASp
ASTp
NASs
ASTs
NASk
ASTk
P < 0.01
P < 0.01
P < 0.01
P < 0.01
P < 0.01

## Slide 3
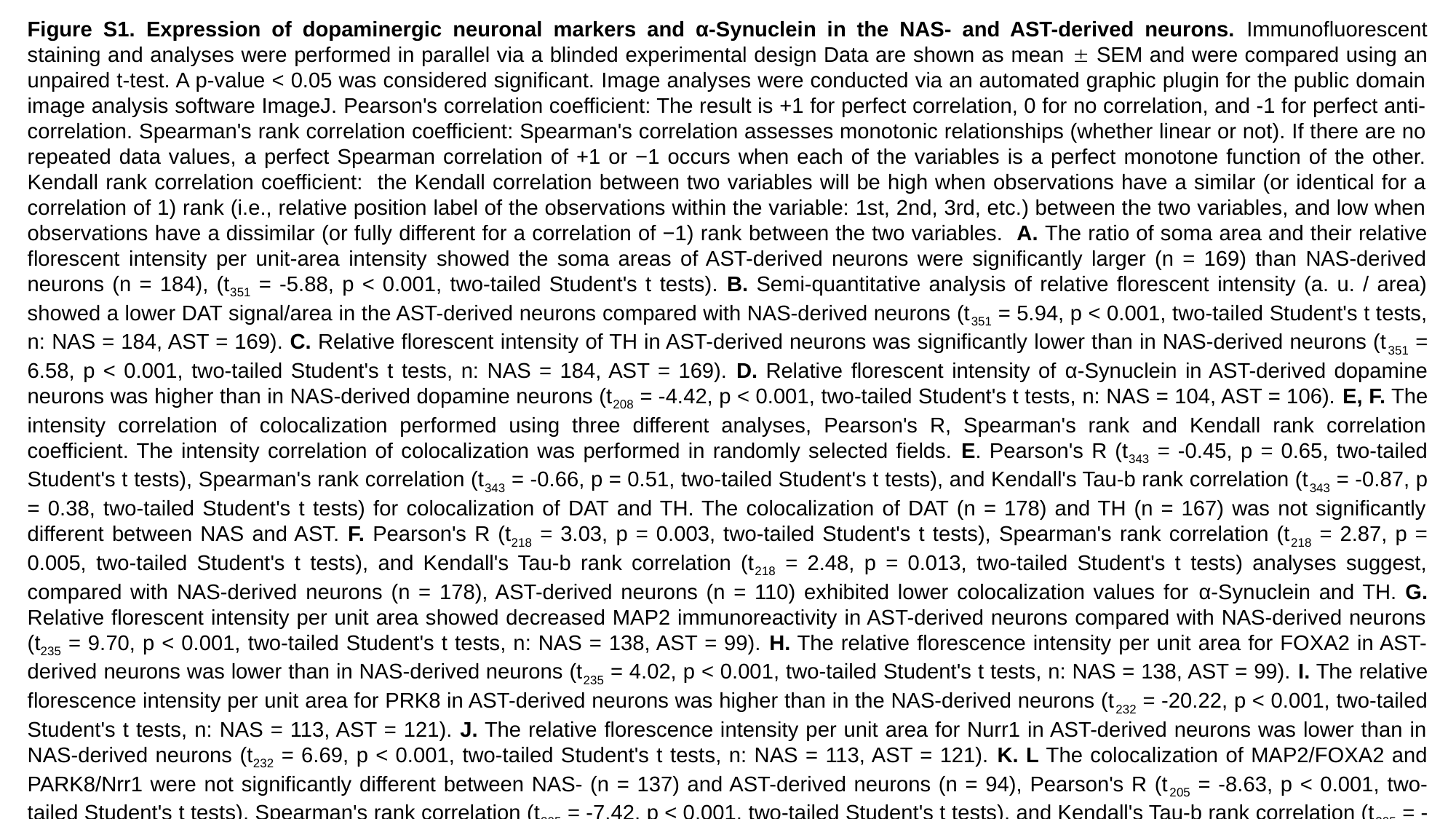

Figure S1. Expression of dopaminergic neuronal markers and α-Synuclein in the NAS- and AST-derived neurons. Immunofluorescent staining and analyses were performed in parallel via a blinded experimental design Data are shown as mean ± SEM and were compared using an unpaired t-test. A p-value < 0.05 was considered significant. Image analyses were conducted via an automated graphic plugin for the public domain image analysis software ImageJ. Pearson's correlation coefficient: The result is +1 for perfect correlation, 0 for no correlation, and -1 for perfect anti-correlation. Spearman's rank correlation coefficient: Spearman's correlation assesses monotonic relationships (whether linear or not). If there are no repeated data values, a perfect Spearman correlation of +1 or −1 occurs when each of the variables is a perfect monotone function of the other. Kendall rank correlation coefficient: the Kendall correlation between two variables will be high when observations have a similar (or identical for a correlation of 1) rank (i.e., relative position label of the observations within the variable: 1st, 2nd, 3rd, etc.) between the two variables, and low when observations have a dissimilar (or fully different for a correlation of −1) rank between the two variables. A. The ratio of soma area and their relative florescent intensity per unit-area intensity showed the soma areas of AST-derived neurons were significantly larger (n = 169) than NAS-derived neurons (n = 184), (t351 = -5.88, p < 0.001, two-tailed Student's t tests). B. Semi-quantitative analysis of relative florescent intensity (a. u. / area) showed a lower DAT signal/area in the AST-derived neurons compared with NAS-derived neurons (t351 = 5.94, p < 0.001, two-tailed Student's t tests, n: NAS = 184, AST = 169). C. Relative florescent intensity of TH in AST-derived neurons was significantly lower than in NAS-derived neurons (t351 = 6.58, p < 0.001, two-tailed Student's t tests, n: NAS = 184, AST = 169). D. Relative florescent intensity of α-Synuclein in AST-derived dopamine neurons was higher than in NAS-derived dopamine neurons (t208 = -4.42, p < 0.001, two-tailed Student's t tests, n: NAS = 104, AST = 106). E, F. The intensity correlation of colocalization performed using three different analyses, Pearson's R, Spearman's rank and Kendall rank correlation coefficient. The intensity correlation of colocalization was performed in randomly selected fields. E. Pearson's R (t343 = -0.45, p = 0.65, two-tailed Student's t tests), Spearman's rank correlation (t343 = -0.66, p = 0.51, two-tailed Student's t tests), and Kendall's Tau-b rank correlation (t343 = -0.87, p = 0.38, two-tailed Student's t tests) for colocalization of DAT and TH. The colocalization of DAT (n = 178) and TH (n = 167) was not significantly different between NAS and AST. F. Pearson's R (t218 = 3.03, p = 0.003, two-tailed Student's t tests), Spearman's rank correlation (t218 = 2.87, p = 0.005, two-tailed Student's t tests), and Kendall's Tau-b rank correlation (t218 = 2.48, p = 0.013, two-tailed Student's t tests) analyses suggest, compared with NAS-derived neurons (n = 178), AST-derived neurons (n = 110) exhibited lower colocalization values for α-Synuclein and TH. G. Relative florescent intensity per unit area showed decreased MAP2 immunoreactivity in AST-derived neurons compared with NAS-derived neurons (t235 = 9.70, p < 0.001, two-tailed Student's t tests, n: NAS = 138, AST = 99). H. The relative florescence intensity per unit area for FOXA2 in AST-derived neurons was lower than in NAS-derived neurons (t235 = 4.02, p < 0.001, two-tailed Student's t tests, n: NAS = 138, AST = 99). I. The relative florescence intensity per unit area for PRK8 in AST-derived neurons was higher than in the NAS-derived neurons (t232 = -20.22, p < 0.001, two-tailed Student's t tests, n: NAS = 113, AST = 121). J. The relative florescence intensity per unit area for Nurr1 in AST-derived neurons was lower than in NAS-derived neurons (t232 = 6.69, p < 0.001, two-tailed Student's t tests, n: NAS = 113, AST = 121). K. L The colocalization of MAP2/FOXA2 and PARK8/Nrr1 were not significantly different between NAS- (n = 137) and AST-derived neurons (n = 94), Pearson's R (t205 = -8.63, p < 0.001, two-tailed Student's t tests), Spearman's rank correlation (t205 = -7.42, p < 0.001, two-tailed Student's t tests), and Kendall's Tau-b rank correlation (t205 = -7.40, p < 0.001, two-tailed Student's t tests).

## Slide 4
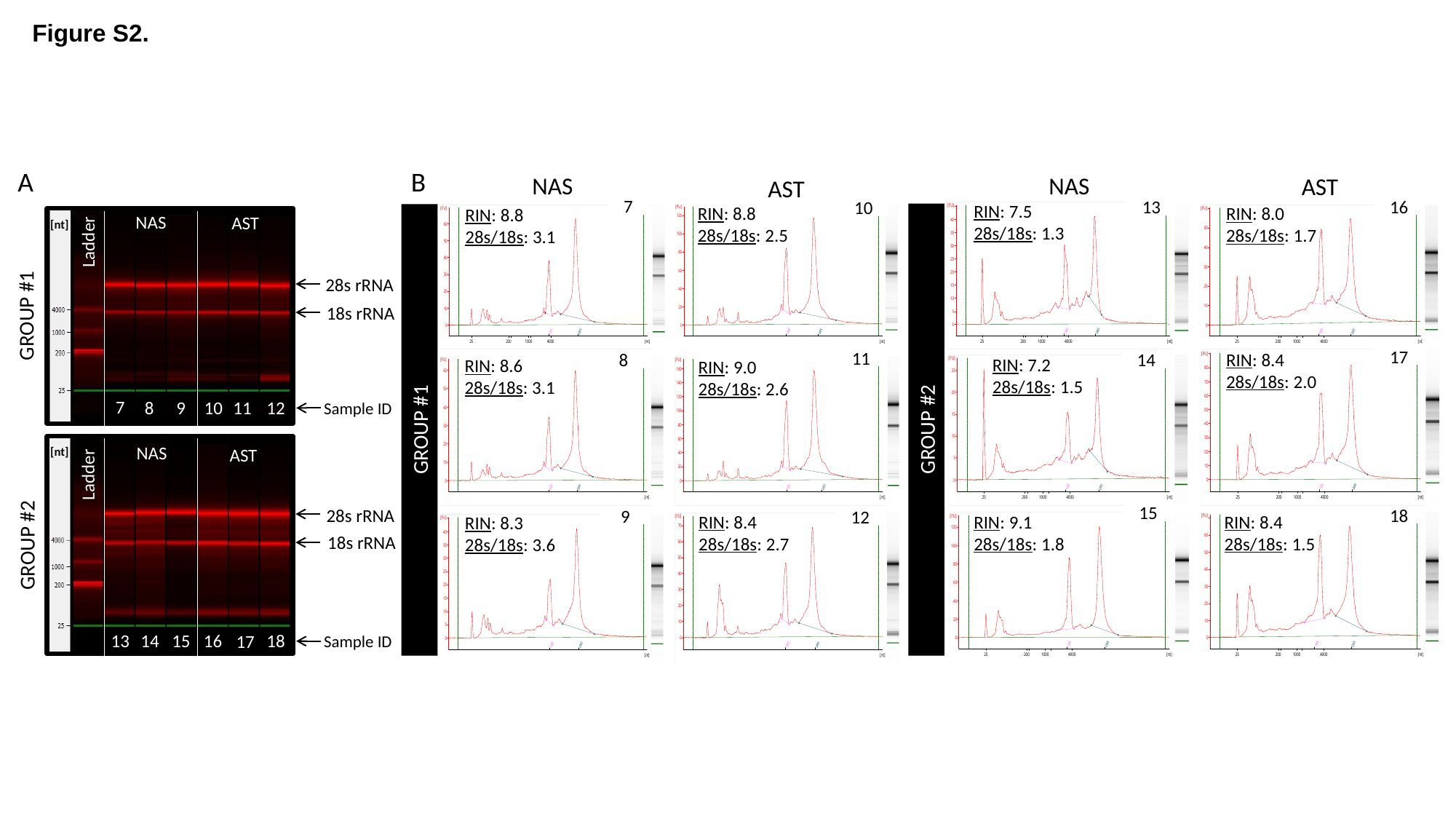

Figure S2.
B
A
NAS
AST
RIN: 8.8
28s/18s: 2.5
RIN: 8.8
28s/18s: 3.1
RIN: 8.6
28s/18s: 3.1
RIN: 9.0
28s/18s: 2.6
RIN: 8.4
28s/18s: 2.7
RIN: 8.3
28s/18s: 3.6
NAS
AST
RIN: 7.5
28s/18s: 1.3
RIN: 8.0
28s/18s: 1.7
RIN: 8.4
28s/18s: 2.0
RIN: 7.2
28s/18s: 1.5
RIN: 9.1
28s/18s: 1.8
RIN: 8.4
28s/18s: 1.5
NAS
AST
[nt]
Ladder
28s rRNA
18s rRNA
GROUP #1
7
9
8
10
11
12
NAS
[nt]
AST
Ladder
28s rRNA
18s rRNA
GROUP #2
16
18
15
13
14
17
GROUP #2
GROUP #1
7
16
13
10
17
11
14
8
15
18
9
12
Sample ID
Sample ID

## Slide 5
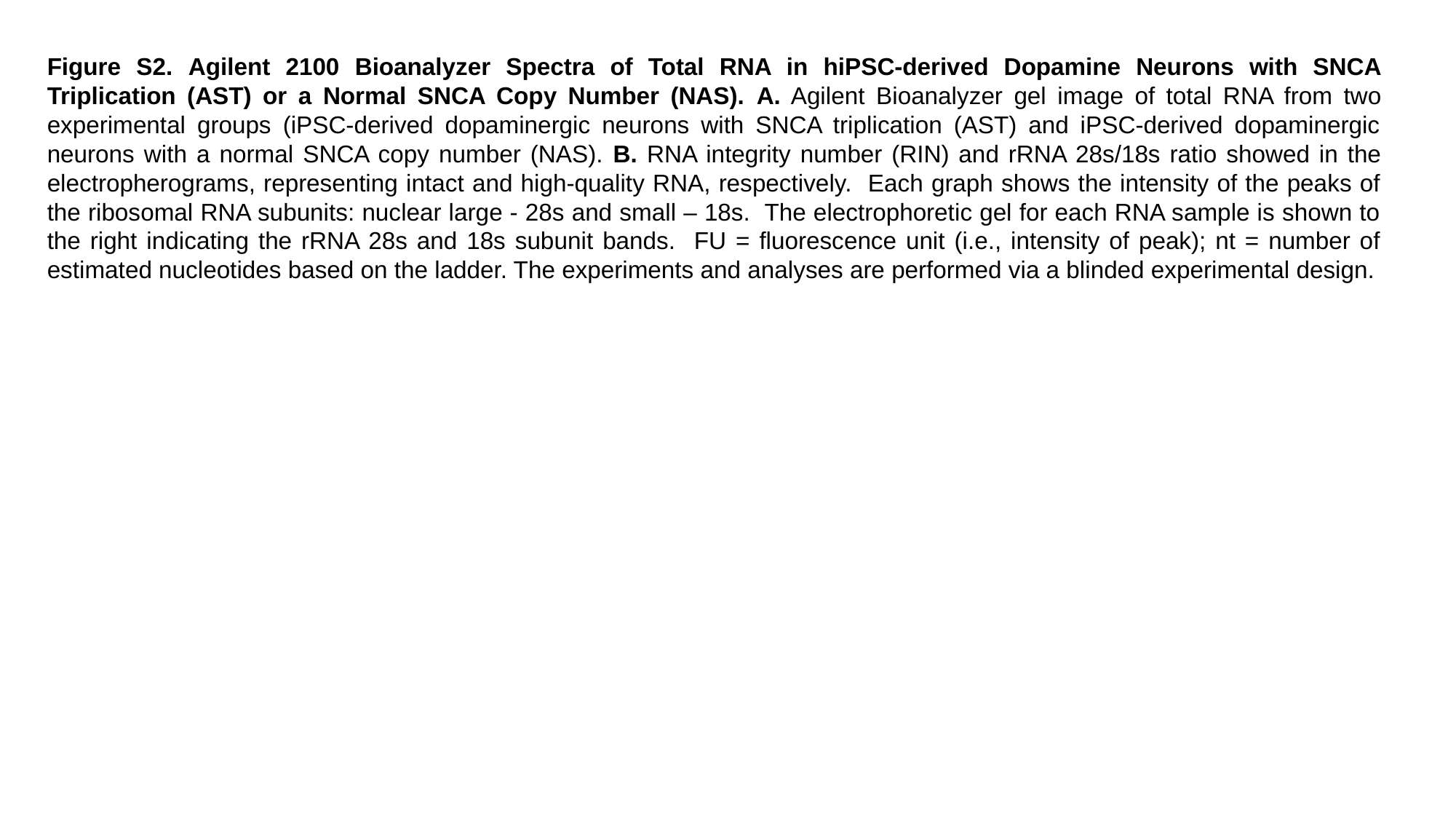

Figure S2. Agilent 2100 Bioanalyzer Spectra of Total RNA in hiPSC-derived Dopamine Neurons with SNCA Triplication (AST) or a Normal SNCA Copy Number (NAS). A. Agilent Bioanalyzer gel image of total RNA from two experimental groups (iPSC-derived dopaminergic neurons with SNCA triplication (AST) and iPSC-derived dopaminergic neurons with a normal SNCA copy number (NAS). B. RNA integrity number (RIN) and rRNA 28s/18s ratio showed in the electropherograms, representing intact and high-quality RNA, respectively. Each graph shows the intensity of the peaks of the ribosomal RNA subunits: nuclear large - 28s and small – 18s. The electrophoretic gel for each RNA sample is shown to the right indicating the rRNA 28s and 18s subunit bands. FU = fluorescence unit (i.e., intensity of peak); nt = number of estimated nucleotides based on the ladder. The experiments and analyses are performed via a blinded experimental design.

## Slide 6
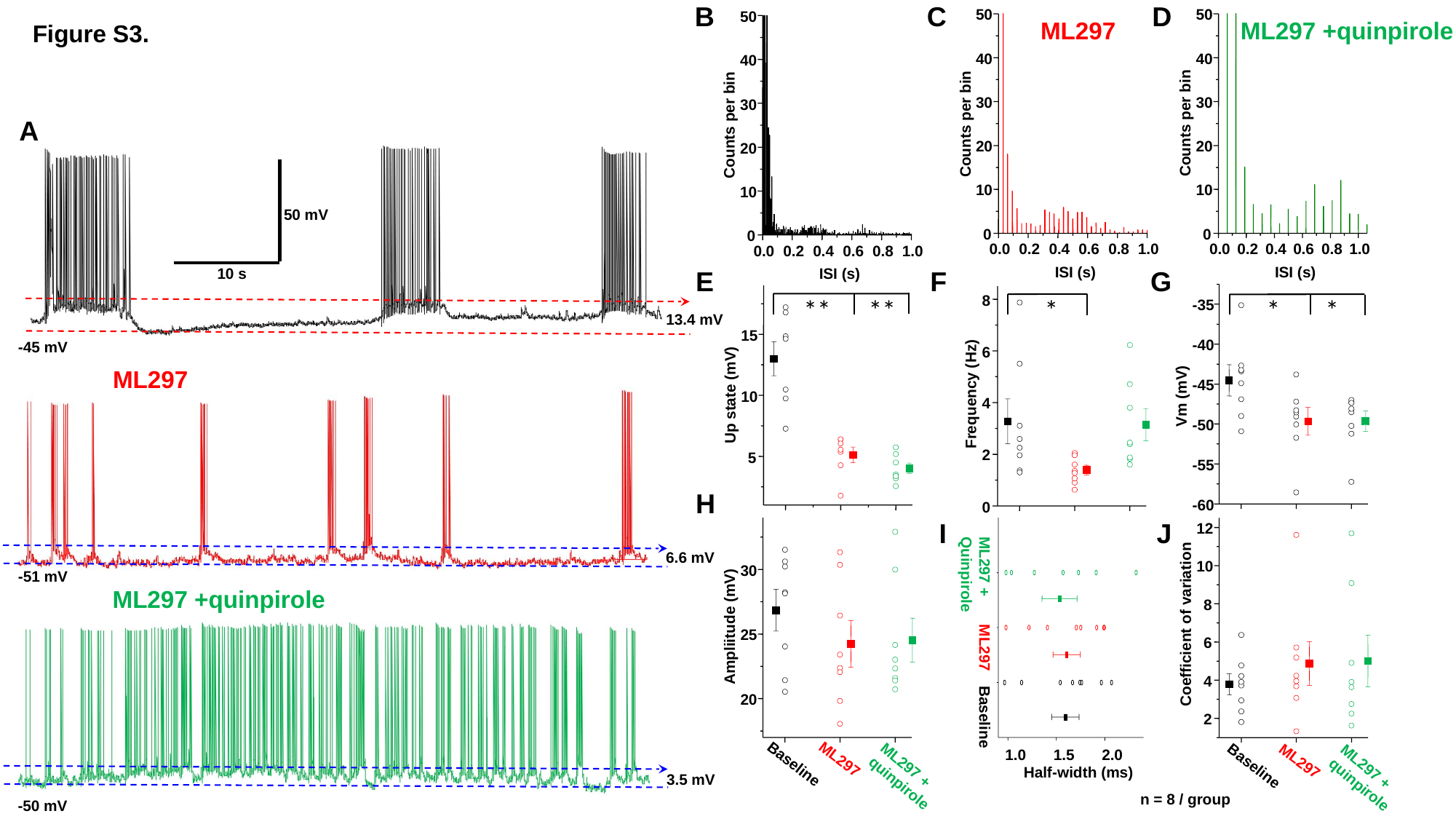

B
C
D
50
40
30
Counts per bin
20
10
0
0.0
0.2
0.4
0.6
0.8
1.0
ISI (s)
50
40
30
Counts per bin
20
10
0
0.0
0.2
0.4
0.6
0.8
1.0
ISI (s)
50
40
30
Counts per bin
20
10
0
0.0
0.2
0.4
0.6
0.8
1.0
ISI (s)
Figure S3.
ML297
ML297 +quinpirole
A
50 mV
10 s
E
F
G
-35
-40
-45
Vm (mV)
-50
-55
-60
15
Up state (mV)
10
5
8
6
Frequency (Hz)
4
2
0
**
**
*
*
*
13.4 mV
-45 mV
ML297
H
I
J
ML297 +
Quinpirole
ML297
Baseline
1.0
1.5
2.0
Half-width (ms)
30
Ampliitude (mV)
25
20
ML297
Baseline
ML297 +
quinpirole
12
10
8
Coefficient of variation
6
4
2
ML297
Baseline
ML297 +
quinpirole
6.6 mV
-51 mV
ML297 +quinpirole
3.5 mV
n = 8 / group
-50 mV

## Slide 7
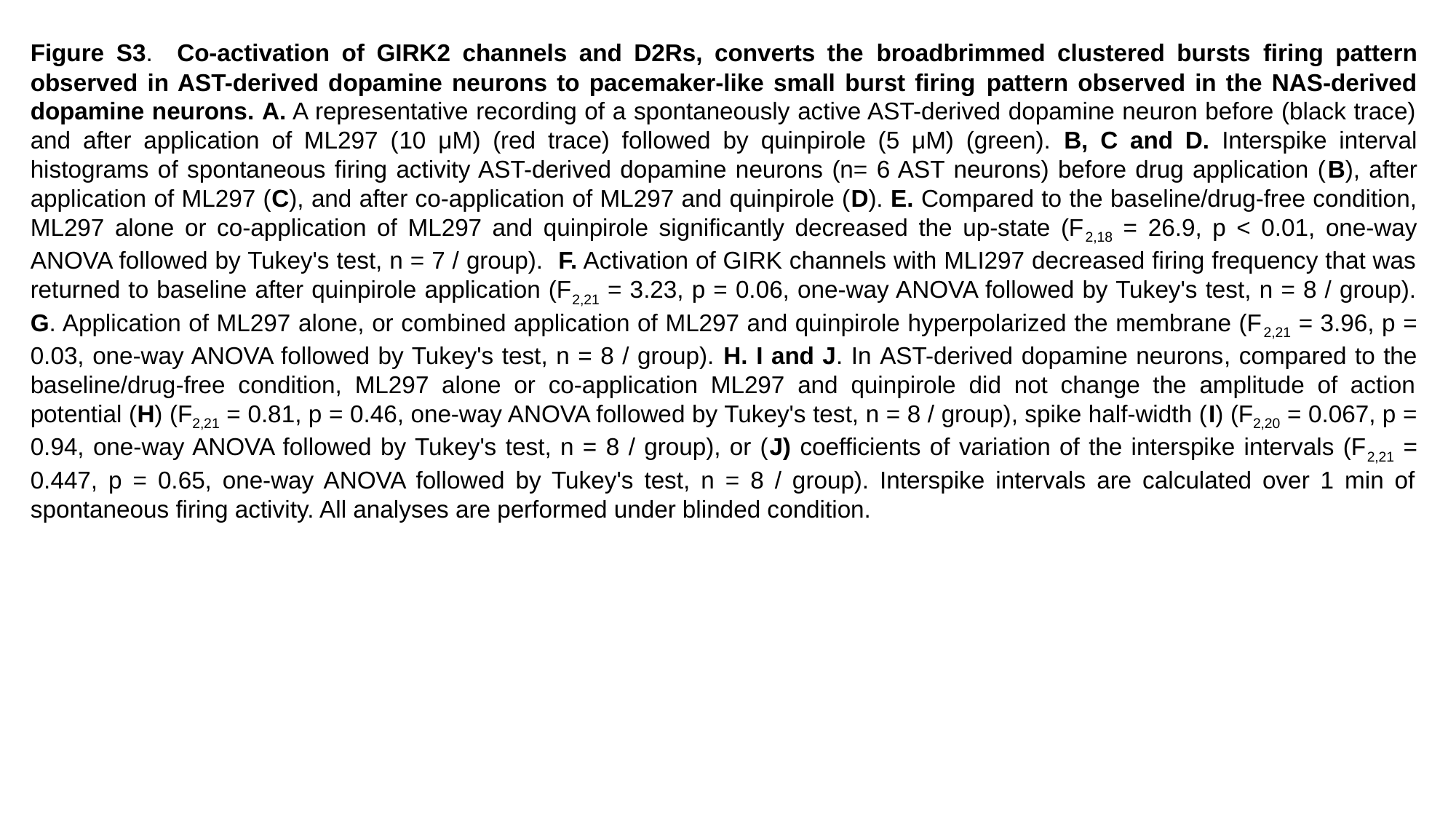

Figure S3. Co-activation of GIRK2 channels and D2Rs, converts the broadbrimmed clustered bursts firing pattern observed in AST-derived dopamine neurons to pacemaker-like small burst firing pattern observed in the NAS-derived dopamine neurons. A. A representative recording of a spontaneously active AST-derived dopamine neuron before (black trace) and after application of ML297 (10 μM) (red trace) followed by quinpirole (5 μM) (green). B, C and D. Interspike interval histograms of spontaneous firing activity AST-derived dopamine neurons (n= 6 AST neurons) before drug application (B), after application of ML297 (C), and after co-application of ML297 and quinpirole (D). E. Compared to the baseline/drug-free condition, ML297 alone or co-application of ML297 and quinpirole significantly decreased the up-state (F2,18 = 26.9, p < 0.01, one-way ANOVA followed by Tukey's test, n = 7 / group). F. Activation of GIRK channels with MLI297 decreased firing frequency that was returned to baseline after quinpirole application (F2,21 = 3.23, p = 0.06, one-way ANOVA followed by Tukey's test, n = 8 / group). G. Application of ML297 alone, or combined application of ML297 and quinpirole hyperpolarized the membrane (F2,21 = 3.96, p = 0.03, one-way ANOVA followed by Tukey's test, n = 8 / group). H. I and J. In AST-derived dopamine neurons, compared to the baseline/drug-free condition, ML297 alone or co-application ML297 and quinpirole did not change the amplitude of action potential (H) (F2,21 = 0.81, p = 0.46, one-way ANOVA followed by Tukey's test, n = 8 / group), spike half-width (I) (F2,20 = 0.067, p = 0.94, one-way ANOVA followed by Tukey's test, n = 8 / group), or (J) coefficients of variation of the interspike intervals (F2,21 = 0.447, p = 0.65, one-way ANOVA followed by Tukey's test, n = 8 / group). Interspike intervals are calculated over 1 min of spontaneous firing activity. All analyses are performed under blinded condition.

## Slide 8
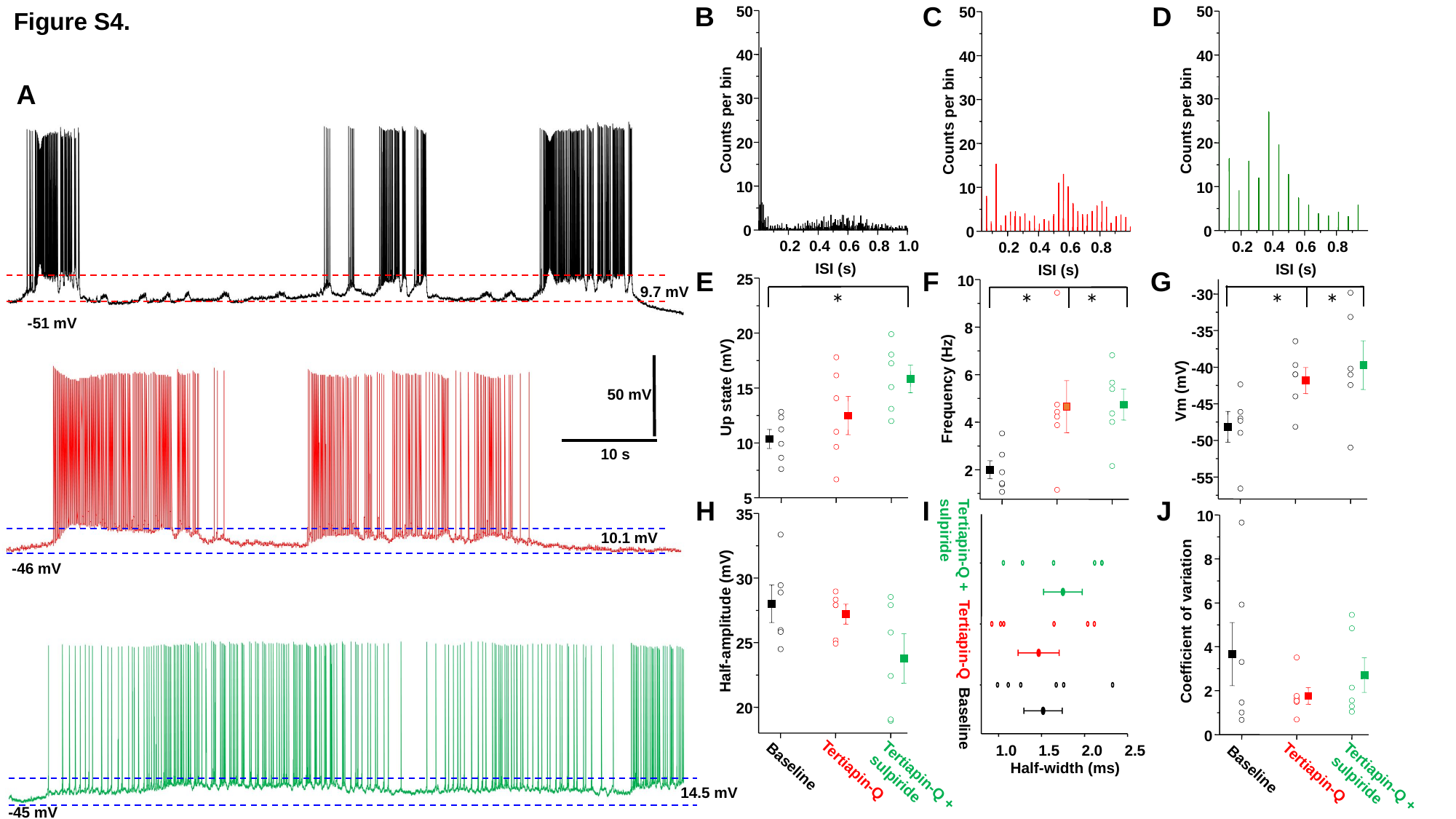

B
C
D
Figure S4.
50
40
30
Counts per bin
20
10
0
0.2
0.4
0.6
0.8
1.0
ISI (s)
50
40
30
Counts per bin
20
10
0
0.2
0.4
0.6
0.8
ISI (s)
50
40
30
Counts per bin
20
10
0
0.2
0.4
0.6
0.8
ISI (s)
A
E
F
G
25
20
Up state (mV)
15
10
5
10
8
6
Frequency (Hz)
4
2
9.7 mV
-30
-35
-40
Vm (mV)
-45
-50
-55
*
*
*
*
*
-51 mV
50 mV
10 s
H
I
J
Tertiapin-Q + sulpiride
Tertiapin-Q
Baseline
1.0
1.5
2.0
2.5
Half-width (ms)
35
30
Half-amplitude (mV)
25
20
Baseline
Tertiapin-Q
Tertiapin-Q +
sulpiride
10
8
6
Coefficient of variation
4
2
0
Baseline
Tertiapin-Q
Tertiapin-Q +
sulpiride
10.1 mV
-46 mV
14.5 mV
-45 mV

## Slide 9
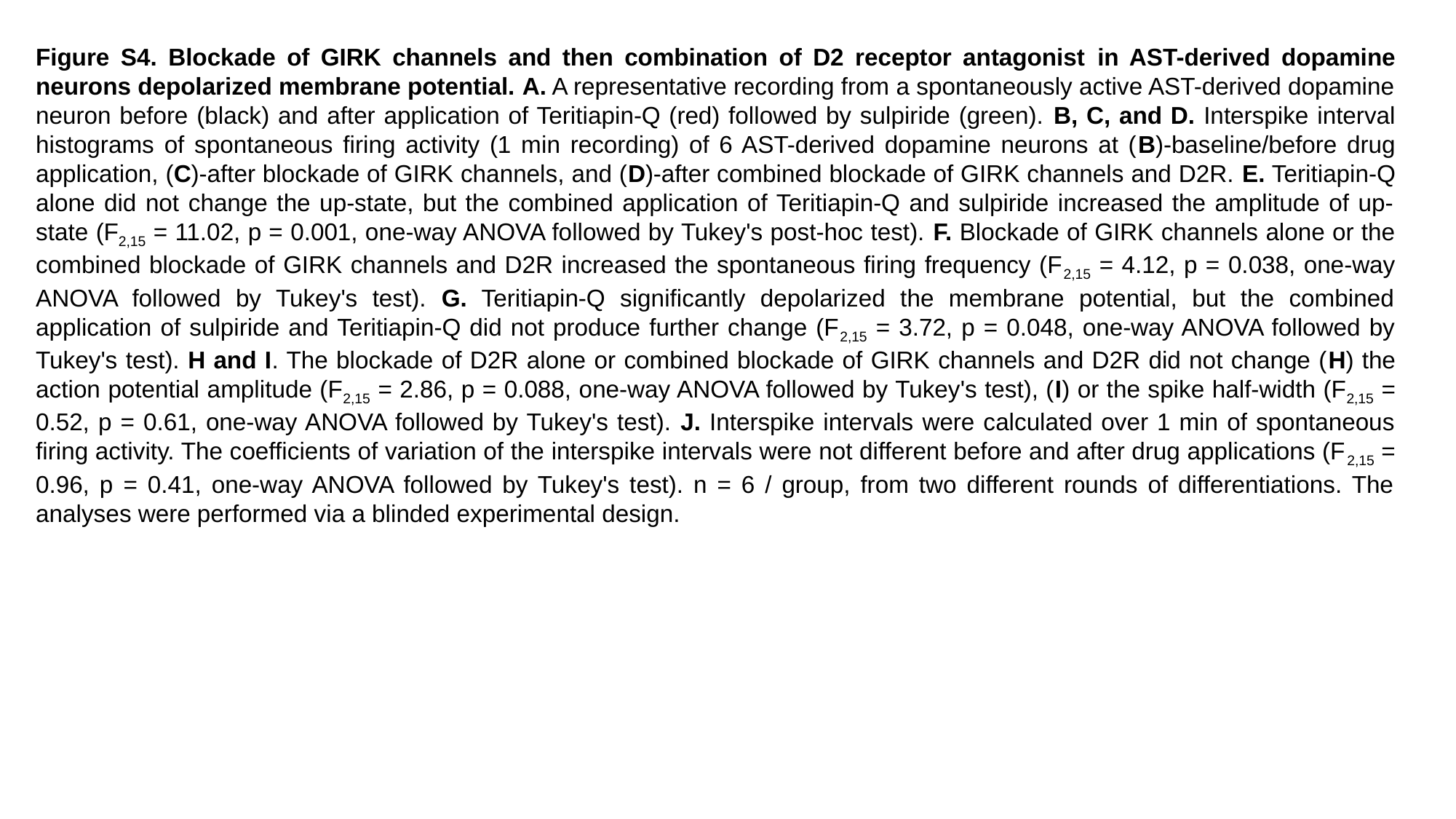

Figure S4. Blockade of GIRK channels and then combination of D2 receptor antagonist in AST-derived dopamine neurons depolarized membrane potential. A. A representative recording from a spontaneously active AST-derived dopamine neuron before (black) and after application of Teritiapin-Q (red) followed by sulpiride (green). B, C, and D. Interspike interval histograms of spontaneous firing activity (1 min recording) of 6 AST-derived dopamine neurons at (B)-baseline/before drug application, (C)-after blockade of GIRK channels, and (D)-after combined blockade of GIRK channels and D2R. E. Teritiapin-Q alone did not change the up-state, but the combined application of Teritiapin-Q and sulpiride increased the amplitude of up-state (F2,15 = 11.02, p = 0.001, one-way ANOVA followed by Tukey's post-hoc test). F. Blockade of GIRK channels alone or the combined blockade of GIRK channels and D2R increased the spontaneous firing frequency (F2,15 = 4.12, p = 0.038, one-way ANOVA followed by Tukey's test). G. Teritiapin-Q significantly depolarized the membrane potential, but the combined application of sulpiride and Teritiapin-Q did not produce further change (F2,15 = 3.72, p = 0.048, one-way ANOVA followed by Tukey's test). H and I. The blockade of D2R alone or combined blockade of GIRK channels and D2R did not change (H) the action potential amplitude (F2,15 = 2.86, p = 0.088, one-way ANOVA followed by Tukey's test), (I) or the spike half-width (F2,15 = 0.52, p = 0.61, one-way ANOVA followed by Tukey's test). J. Interspike intervals were calculated over 1 min of spontaneous firing activity. The coefficients of variation of the interspike intervals were not different before and after drug applications (F2,15 = 0.96, p = 0.41, one-way ANOVA followed by Tukey's test). n = 6 / group, from two different rounds of differentiations. The analyses were performed via a blinded experimental design.

## Slide 10
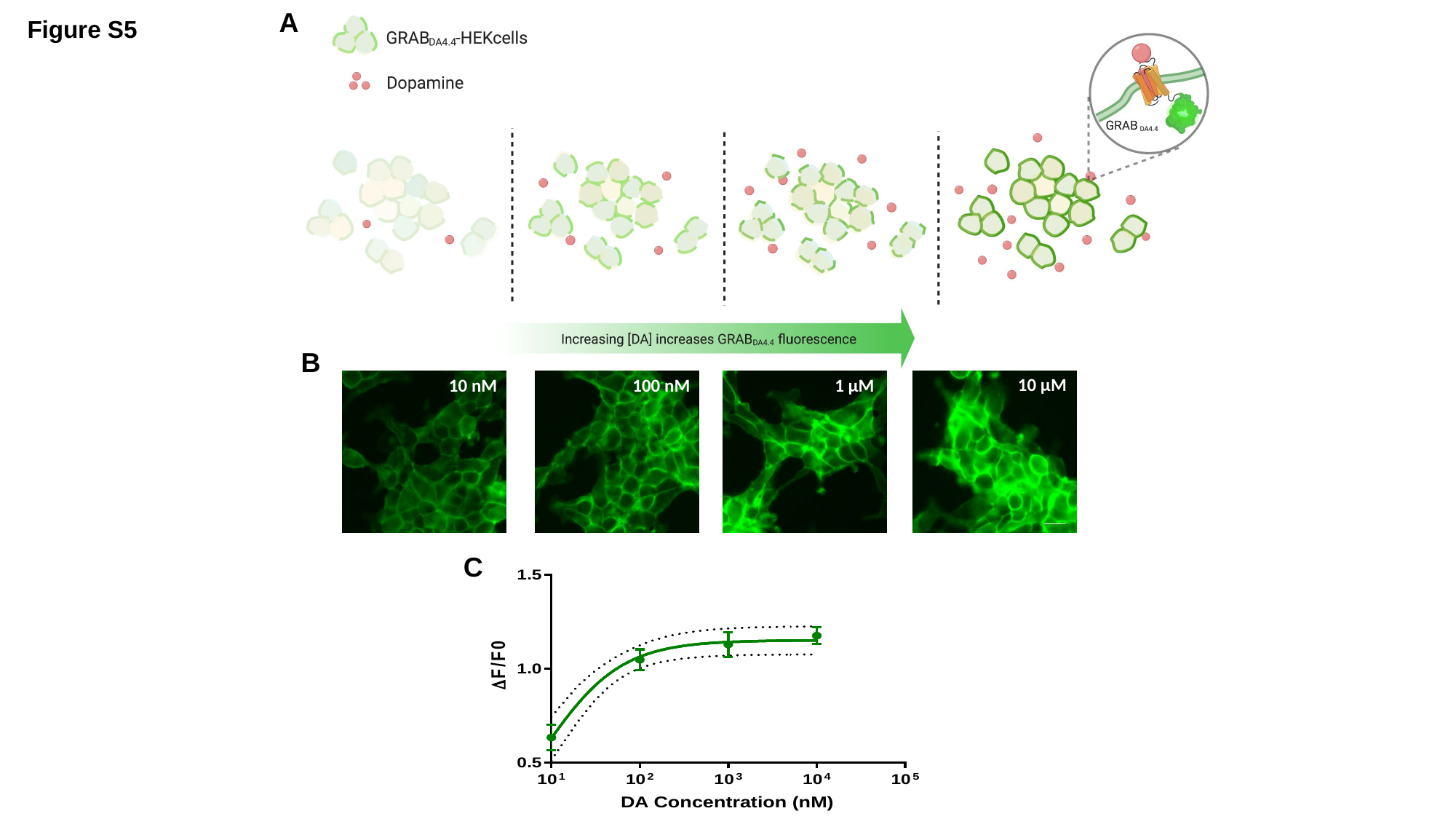

A
Figure S5
B
10 µM
10 nM
100 nM
1 µM
C

## Slide 11
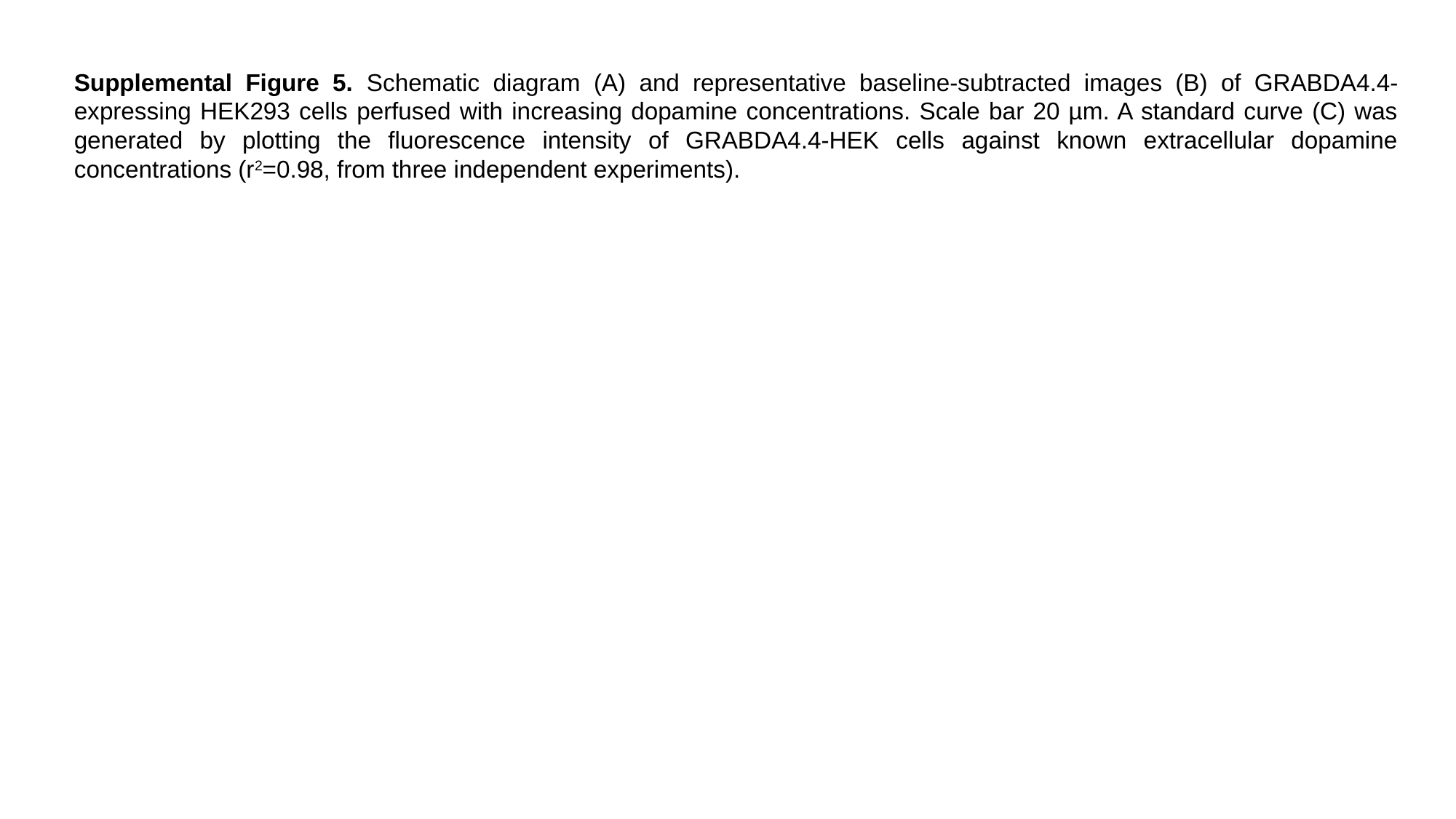

Supplemental Figure 5. Schematic diagram (A) and representative baseline-subtracted images (B) of GRABDA4.4-expressing HEK293 cells perfused with increasing dopamine concentrations. Scale bar 20 µm. A standard curve (C) was generated by plotting the fluorescence intensity of GRABDA4.4-HEK cells against known extracellular dopamine concentrations (r2=0.98, from three independent experiments).
