# Supplementary material for: In Parkinson's patient-derived dopamine neurons, the triplication of α-synuclein locus induces distinctive firing pattern by impeding D2 receptor autoinhibition
Source: Acta Neuropathol Commun. 2021 Jun 7;9:107. doi: 10.1186/s40478-021-01203-9 (PMC8185945; doi:10.1186/s40478-021-01203-9)
Supplement: Supplementary file 2 — Additional file 2: Results for supplemental data. [file 40478_2021_1203_MOESM2_ESM.docx]

**Supplemental data**

**In Parkinson's patient-derived dopamine neurons, the triplication of α-synuclein locus induces distinctive firing pattern by impeding D2 receptor autoinhibition**

Min Lin^1^, Phillip M. Mackie^1^, Fatima Shaerzadeh^1^, Joyonna Gamble-George^1^, Douglas R. Miller^1^, Chris J. Martyniuk^2^, Habibeh Khoshbouei^1^

^1^Department of Neuroscience, University of Florida, Gainesville, FL 32611

^2^Environmental and Human Toxicology, University of Florida Genetics Institute, Interdisciplinary Program in Biomedical Sciences Neuroscience, College of Veterinary Medicine, University of Florida, Gainesville, FL, 32611 USA

**Quantifications for the** **expression of human dopaminergic neuron markers.** With methodologies described in previous reports (Burgess et al., 2010, Cvetkovska et al., 2013, Viotti et al., 2014, Lo et al., 2015), we performed quantifications at day 36 of differentiation. For these analyses, the average intensity was divided by the soma area and shown as relative intensity. As shown in Supplement Figure 1, the area of cell bodies in AST-derived DA neurons were significantly larger (742.8 ± 17.3 μm^2^, n = 169) than NAS-derived DA neurons (617.6 ± 13.5 μm^2^, p < 0.001, n = 184, Suppl. Fig 1A). Gray value of florescence was divided by cell body area as relative intensity to determine the expression of neural markers. Compared with NAS-derived DA neurons (0.26 ± 0.007 a.u. / μm^2^, n = 184), DAT was significantly reduced in expression in AST-derived DA neurons (0.20 ± 0.005 a.u. / μm^2^, n = 169, p < 0.001, Suppl. Fig 1B). TH was also significantly reduced in expression in AST-derived DA neurons (0.16 ± 0.005 a.u. / μm^2^, n = 169) compared with NAS (0.22 ± 0.007 a.u. / μm^2^, p < 0.001, n = 184, Suppl. Fig 1C). In contrast, α-synuclein exhibited higher expression levels in AST-derived DA neurons (0.22 ± 0.007 a.u. / μm^2^, n = 108) compared with NAS-derived DA neurons (0.18 ± 0.006 a.u. / μm^2^, p < 0.001, n = 111, Suppl. Fig 1D). The intensity correlation of colocalization was performed using three different analysis. Pearson's, Spearman's rank and Kendall rank correlation coefficient analysis revealed that DAT equally colocalized with TH between NAS- and AST-derived DA neurons (Suppl. Fig 1E). In contrast, α-synuclein also positively correlated with TH in both AST and NAS, but AST showed lower overlap between α-synuclein and TH expression compared to NAS (Suppl. Fig 1F). The relative FOXA2 (0.30 ± 0.01 a.u. / μm^2^, n = 138) and MAP2 intensities (0.23 ± 0.01 a.u. / μm^2^, n = 99) were lower in AST-derived dopamine neurons compared to NAS-derived dopamine neurons (FOXA2: 0.39 ± 0.02 a.u. / μm^2^, n = 138; MAP2: 0.51 ± 0.02 a.u. / μm^2^, p < 0.001, n = 138, Suppl. Figure 1G – 1H). (NAS: 0.49 ± 0.02 a.u. / μm^2^ vs. 0.34 ± 0.01 a.u. / μm^2^, n: NAS = 113, p < 0.001, AST = 121, Suppl. Figure 1G – 1H). In addition to FOXA2 and MAP2, we also observed lower expression of another midbrain marker Nurr1 in AST-derived DA neurons (NAS: 0.49 ± 0.02 a.u. / μm^2^ vs. AST: 0.34 ± 0.01 a.u. / μm^2^, n: NAS = 113, p < 0.001, AST = 121, Suppl. Figure 1J). Consistent with previous studies (Kitada et al., 1998, Shimura et al., 2001), robust expression of parkin was detected in AST-derived dopamine neurons (0.44 ± 0.02 a.u. / μm^2^, n = 121); whereas a much smaller relative intensity value was observed in NAS-derived dopamine neurons (0.06 ± 0.00 a.u. / μm^2^, p < 0.001, n = 113, Suppl. Figure 1I). To determine the degree of the overlap of fluorescently labeled molecules within the neurons, we employed the frequently described intensity correlation of colocalization analysis (Bolte and Cordelières 2006, Dunn et al., 2011, Wang et al., 2018) using three different statistical comparisons. Examinations of Pearson's, Spearman's rank and Kendall rank correlation coefficient revealed comparable colocalization/overlap values of FOXA2 and MAP2 signals between NAS- and AST-derived dopamine neurons (Suppl. Figure 1K). Unlike the AST-derived dopamine neurons, the basal PARK8 expression was low in the NAS-derived dopamine neurons hence a near zero colocalization coefficient for PARK8 and Nurr1 signals in these neurons (Suppl. Figure 1L).

**Combined application of dopamine D2 receptor agonist and G-protein-activated inwardly rectifying potassium channels (GIRK2) convert the firing pattern of AST-derived dopamine neurons to NAS-like firing activity.** GIRK channels are expressed in the dopamine neurons of substantia nigra and ventral tegmental area of rodents (Karschin et al., 1996) and human (Mendez et al., 2005). In dopamine neurons, D2R autoinhibition is coupled to GIRK2 channels activity (Lacey et al., 1987; Kim et al., 1995), and activation of D2R-coupled GIRK channels inhibits dopamine neurons (Cruz et al., 2004; Labouèbe et al., 2007; Arora et al., 2010), leading to neuronal hyperpolarization and reducing membrane excitability (Lacey et al., 1987; Beckstead et al., 2004; Lüscher and Slesinger 2010). In the AST-derived dopamine neurons the RNA for KCNJ6G, the gene encoding GIRK2 (Figure 2B) and GIRK2 immunostaining (Figure 9E, 9F) are decreased; therefore, we investigated whether GIRK2 activation reinstates the firing pattern of AST-derived dopamine neurons to NAS-like firing activity. Similar to quinpirole-activation of D2R (Figures 5 and 6), a GIRK2 channel activator such as ML297 reduces the magnitude of up-state and firing frequency. As shown in Suppl. Figure 3C, bath application of ML297 (10 μM) increased the interspike interval of AST-derived dopamine neurons (baseline: 570.9 ± 98.4 ms vs. ML297: 1049.9 ± 142.9 ms, p = 0.01, n = 8 / group). Co-application of ML297 and quinpirole (Suppl. Figure 3D) also increased the interspike interval compared with the baseline (1691.0 ± 419.9 ms, Suppl. Figure 3D), that was similar to quinpirole only treatment. ML297 suppressed the amplitude of up-state (baseline: 12.99 ± 1.39 mV vs. ML297: 5.11 ± 0.64 mV), but the fold reduction in the up-state was not different from ML297 + quinpirole co-application (Suppl. Figure 3E, 4.01 ± 0.23 mV). ML297 reduced the spontaneous firing frequency (Suppl. Figure 3F, baseline: 3.25 ± 0.87 Hz, ML297: 1.36 ± 0.19 Hz) and hyperpolarized membrane potentials (baseline: -44.5 ± 2.0 mV, ML297: -49.65 ± 1.74 mV (Suppl. Figure 3G).

Importantly, co-application of ML297 and quinpirole converted the broadbrimmed firing burst of AST-derived dopamine neurons to pacemaker-like small burst (Suppl. Figure 3A, lower panel). Surprisingly, the firing frequency (Suppl. Figure 3F, 3.11 ± 0.62 Hz), action potential amplitude (Suppl. Figure 3H) or half-width (Suppl. Figure 3I) did not decrease, suggesting GIRK is the downstream mediator of D2R activity in these neurons. These data suggest although individually D2R agonist or GIRK activation reduces the burst clusters, firing rate and up-state amplitude in AST-derived dopamine neurons, co-administration of D2R and GIRK activators also produces a NAS-like firing pattern.

**Application of GIRK channel blocker and then plus D2 receptor antagonist in the AST-derived dopamine neurons depolarized membrane potential and increased firing frequency.** GIRK activation hyperpolarizes the membrane and plays a fundamental role in regulation of neuronal excitability (Suppl. Figure 3, Signorini et al., 1997). To further investigate the functional relationship between D2R and GIRK channels and their contribution to the firing pattern of AST-derived dopamine neurons, we studied the effects of blockade of GIRK channels or combined blockade of GIRK and D2R on the intrinsic firing behavior in these neurons (Suppl. Figure 4). The blockade of GIRK channels (Suppl. Figure 4B) or concomitant blockade of GIRK and D2R (Suppl. Figure 4C) increased the distribution range of interspike intervals in the AST-derived dopamine neurons. it induced a large size of broadbrimmed firing burst (Suppl. Figure 4A). Blockade of GIRK channels alone did not affect the amplitude of up-state (Suppl. Figure 4E), in contrast, combined Tertiapin-Q, a GIRK channel blocker and sulpiride mildly enhanced up-state. (Suppl. Figure 4A bottom trace, 4E). As expected Tertiapin-Q depolarized membrane potential (baseline: -48.06 ± 2.11 mV vs. Tertiapin-Q: -41.7 ± 1.8 mV), whereas combined Tertiapin-Q and sulpiride (-39.6 ± 3.3 mV, Suppl. Figure 4G) did not produce additional membrane depolarization suggesting GIRK is the downstream mediator of D2R activation in these neurons. The depolarized membrane potential underlies high firing frequency after application of Tertiapin-Q alone or combined Tertiapin-Q and sulpiride (Suppl. Figure 4F). Tertiapin-Q alone or Tertiapin-Q plus sulpiride did not change action potential amplitude (Suppl. Figure 4H), half-width (Suppl. Figure 4I) and the coefficient of variation of interspike intervals (Suppl. Figure 4J). These data suggest that GIRK channels in AST-derived dopamine neurons regulate the firing pattern and rate through modifying the interspike interval and membrane potential. In contrast, D2R may modulate the firing pattern and rate through regulating the up/down state and membrane potential.
