# Supplementary material for: In Parkinson's patient-derived dopamine neurons, the triplication of α-synuclein locus induces distinctive firing pattern by impeding D2 receptor autoinhibition
Source: Acta Neuropathol Commun. 2021 Jun 7;9:107. doi: 10.1186/s40478-021-01203-9 (PMC8185945; doi:10.1186/s40478-021-01203-9)
Supplement: Supplementary file 3 — Additional file 3: Gene sets enriched for dopamine signaling and processing. [file 40478_2021_1203_MOESM3_ESM.docx]

**Supplemental Table 1.** Gene sets enriched for dopamine signaling and processing. Shown in the table are the number of entities in the network, the number measured, the median change of the network, the p-value, and category.

| **Name** | **# of Entities** | **Expanded # of Entities** | **# of Measured Entities** | **Median change** | **Normalized Score** | **p-value** | **Hit type** |
| --- | --- | --- | --- | --- | --- | --- | --- |
| Dopamine/Gs Expression Targets | 38 | 58 | 28 | 1.29 | -1.81 | 0.0018 | Biomarkers |
| DRD1/5 Expression Targets | 27 | 56 | 21 | 1.20 | -1.66 | 0.0054 | Signal Processing |
| DRD3 -> Dopamine Uptake | 10 | 37 | 18 | 1.25 | -1.64 | 0.011 | Signal Processing |
| Dopamine Mediated Glutamate Release/Uptake Circle in Neuron in Migraine | 22 | 44 | 21 | 1.36 | -1.61 | 0.023 | Biological Process |
| Dopamine Mediated Glutamate Release and Glutamate Uptake Circle | 34 | 59 | 28 | 1.34 | -1.61 | 0.0088 | Disease |
